# Carbon Sequestration in Resin-Tapped Slash Pine (*Pinus elliottii* Engelm.) Subtropical Plantations

**DOI:** 10.3390/biology12020324

**Published:** 2023-02-16

**Authors:** Kelly Cristine da Silva Rodrigues-Honda, Camila Fernanda de Oliveira Junkes, Júlio César de Lima, Vinicius de Abreu Waldow, Fernando Souza Rocha, Tanise Luisa Sausen, Cimélio Bayer, Edson Talamini, Arthur Germano Fett-Neto

**Affiliations:** 1 Center for Biotechnology, Department of Botany, Federal University of Rio Grande do Sul (UFRGS), C.P. 15005, Porto Alegre CEP 91501-970, Brazil; 2 Cidade Universitária, Petróleo Brasileiro—CENPES, Rio de Janeiro CEP 21941-915, Brazil; 3 Cerrados Natural Resources Management and Conservation Unit, Brazilian Agricultural Corporation (EMBRAPA), BR 020—Rodovia Brasília-Fortaleza, Planaltina, Brasília CEP 73310970, Brazil; 4 Plant Ecology and Systematics Laboratory, Regional Integrated University of Alto Uruguai and Missões (URI), Erechim CEP 99700-000, Brazil; 5 Soil Department, Faculty of Agronomy, Federal University of Rio Grande do Sul (UFRGS), Porto Alegre CEP 91540-000, Brazil; 6 Interdisciplinary Center for Studies and Research, Bioeconomics Research Group, Department of Economics and International Relations—DERI, Faculty of Economics—FCE, Universidade Federal do Rio Grande do Sul—UFRGS, Agribusiness—CEPAN, 7712—Bairro Agronomia, Porto Alegre 91540-000, Brazil

**Keywords:** *Pinus elliottii* Engelm., pine resin, slash pine, carbon sequestration, carbon stocks

## Abstract

**Simple Summary:**

Pine forests represent a major source of biomass, including timber and resin. Pine resin constitutes a sustainable source of a myriad of products used in several industrial sectors, such as chemicals, pharmaceuticals, food additives, and biofuels. Every year more than 150,000 tons of resin are tapped from Brazilian plantations. A pine tree can be tapped for resin over several years. Resin is a complex mixture of terpenes, which are carbon-rich molecules. Carbon sequestration in plant biomass is an important tool to remove the greenhouse gas CO_2_ from the atmosphere. Resin extraction from pine plantations has been missing as a component in their carbon budget analyses. This detailed study investigated carbon retention in different tree fractions, including extracted resin, of subtropical coastal slash pine plantations. Significantly higher carbon stock values were recorded in subtropical pine biomass compared to those reported for temperate zones. Resin tapping afforded a considerable annual increment in carbon stocks and should be accounted as a relevant component in sequestration assessments of this element in planted pine forests.

**Abstract:**

Every year more than 150,000 tons of resin used in a myriad of industrial applications are produced by Brazilian plantations of *Pinus elliottii* Engelm. (slash pine), which are also used for timber. A pine tree can be tapped for resin over a period of several years. Resin is a complex mixture of terpenes, which are carbon-rich molecules, presumably influencing pine plantation carbon budgets. A total of 270 trees (overall mean DBH of 22.93 ± 0.11 cm) of 14-, 24-, and 26-year-old stands had their C content measured. Three different treatments (intact, wounded panels, and wounded + chemically stimulated panels, 30 trees each) were applied per site. Above- and belowground biomass, as well as resin yield, were quantified for two consecutive years. Data were statistically evaluated using normality distribution tests, analyses of variance, and mean comparison tests (*p* ≤ 0.05). The highest resin production per tree was recorded in the chemically stimulated 14-year-old stand. Tree dry wood biomass, a major stock of carbon retained in cell wall polysaccharides, ranged from 245.69 ± 11.73 to 349.99 ± 16.73 kg among the plantations. Variations in carbon concentration ranged from 43% to 50% with the lowest percentages in underground biomass. There was no significant difference in lignin concentrations. Soils were acidic (pH 4.3 ± 0.10–5.83 ± 0.06) with low C (from 0.05% to 1.4%). Significantly higher C stock values were recorded in pine biomass compared to those reported for temperate zones. Resin-tapping biomass yielded considerable annual increments in C stocks and should be included as a relevant component in C sequestration assessments of planted pine forests.

## 1. Introduction

The greenhouse effect is a natural phenomenon on Earth, generated and primarily controlled by plants as a function of their regular primary metabolism processes. The three main greenhouse gases of concern are carbon dioxide (CO_2_), nitrous oxide (N_2_O), and methane (CH_4_) [1]. The increase in global atmospheric CO_2_ concentration is currently regarded as one of the major factors accelerating the greenhouse effect. According to the established climate change models, it is estimated that increased CO_2_ levels cause faster ozone (O_3_) layer depletion and rising temperatures with relevant consequences on a global scale [2,3]. The imbalanced progress of this natural process, in part attributed to anthropogenic activities, may be mitigated by increasing afforestation [4,5,6,7].

Forests function as carbon sinks [6,8] by fixing atmospheric carbon into both timber and nonwood-derived subproducts, as well as in soils [3,9,10]. Particularly in coniferous (Division Pinophyta, e.g., *Pinus* spp.) forests, carbon storage might be additionally increased by resin (gum resin) production and accumulation in plant tissues. Pine resin is a nonwoody terpene-based biomass that has a high value to the chemical industry [9,11,12]. Resin is also considered a great renewable energy source due to its high calorific (or heating) value, which surpasses that of forest tree woods and its components (e.g., bleached, and unbleached wood pulp) [13]. Despite being constitutively produced in high amounts by some *Pinus* species, its biosynthesis can also be induced by mechanical and chemical treatments [11,14,15,16,17,18,19].

In southern Brazil, roughly 10 million pine trees are currently utilized for producing and exporting gum rosin and turpentine, the two main subproducts of pine resin [20,21]. According to the Brazilian Resin Producers Association (www.aresb.com.br/portal/estatisticas/, accessed 8 November 2022), the Brazilian 2017/2018 crude resin yield was 185,692 tons, most of it (circa 80%) collected from *Pinus elliottii* Engelm. (slash pine) and the remaining 20% was obtained from tropical pines. The nonwood biomass extracted from cultivated pine forests through resin tapping operations might represent an important contribution to the overall carbon fixation budget by these plantations.

Slash pine can reach up to 30 m in height, being characterized by long dark green needles (approximately 15 cm long), scaly reddish-brown bark, dense branching, trunks of 90 to 120 cm in diameter, and cones of approximately 12 cm in length, producing seeds dispersed by the wind. It is native to the coastal and southern U.S.A. In southern Brazil, plantations cover marginal areas of sandy and low-fertility soils along the coast, being explored for both wood and resin. In this habitat, slash pine became an invasive species, requiring some degree of mechanical control to avoid excessive spreading [9]. These pine trees are well known for their profuse resin production, yielding high-quality resin for industrial uses. Their bark and wood are rich in resin ducts that are lined with secretory cells and form a network of canals synthesizing mono, sesqui, and diterpenes [19].

Over the past years, several carbon sequestration estimates have been performed in pine forests growing in temperate, boreal, and Mediterranean Zones. Most of them were carried out based on models built up from biomass allometric regression equations or modeling predictions that might rely on previous local forest information such as inventories data [2,4,6,7,22,23,24,25,26,27,28,29,30,31,32,33,34,35,36,37,38,39] and laser-scanning-based approaches [40,41].

Forest carbon stocks and fluxes are species dependent and affected by several factors, such as forest age [5,6,42,43,44,45,46], genetic background [47], management practices [2,3,5,31,48,49], disturbance effects such as logging or wildfire [26,50], tree density, biomass, regional distribution [25], temperature, precipitation [51], climate zone [6], rotation length, nitrogen deposition, climate conditions [28,52], previous land use [38,53], and soil type [54], among others. Therefore, especially in subtropical areas, assessment of carbon balance based on modeling might lead to a misestimation of actual carbon stocks [55,56]. Furthermore, no considerations have been made on the resin biomass yield in pine stands in terms of its contribution to carbon fixation, especially in regions where *Pinus* spp. are alien species, such as in southern Brazil.

To address this knowledge gap, this work aimed at evaluating carbon content and its distribution among different plant organs, as well as resin biomass contribution to total C in slash pine plantations growing in a subtropical climate. To the best of our knowledge, this is the first report on the destructive and direct assessment of biomass and carbon on pine forests tapped for resin.

## 2. Materials and Methods

### 2.1. Trees, Sites, and Treatments

The study was carried out at the research installations of two Brazilian forest companies (Irani Celulose S.A.—Unidade Resina RS and Âmbar Florestal Ltd.a.). The beginning of trials took place during the fall (May 2009). Fourteen- (A), 24- (B), and 26 (C)-year-old cultivated slash pine (*P. elliottii* Engelm.) stands (not previously tapped for resin removal) were selected in the rural areas of Balneário Pinhal [site A (30°11′17.5″ S, 50°19′23.4″ W), Cidreira [Site B (30°04′16.3″ S, 50°17′07.8″ W)], and Santa Vitória do Palmar [site C (32°54′25.57″ S, 52°32′36.61″ W)], respectively, three cities located on the Atlantic coast of southern Brazil. The climate in these locations is subtropical humid of the Cfa type (Köppen classification). In sites A and B, thinning was performed 10 and 15 years after seedling establishment, respectively, whereas site C had never been thinned at the time of the experiments. Tree densities per hectare were 900 (site A), 600 (site B), and 900 (site C). For tree selection, the first 5 rows of individuals at the margins of the plantations were disregarded to avoid border effects (e.g., potential differences in wind, moisture, and irradiance). Ninety trees randomly distributed within the inner part of the stand were selected in each site based on a DBH (diameter at breast height, i.e., 1.30 m from the soil level) interval previously established (ranging from 22.77 ± 0.09 to 23.48 ± 0.12 cm), according to technical recommendations [57]. Chosen DBH range is considered well-suited for resin tapping and tree number provides statistical robustness for sampling seed-derived plantations. The use of a defined DBH range also eliminated the effect of this parameter on resin yield among trees of the different sites. Trees were distributed in three groups as follows: (IT) intact trees (control treatment) with 30 untreated trees; (BS) bark streak, with 30 mechanically wounded resin tapped trees; and (P) paste, with 30 mechanically wounded and chemically stimulated resin tapped trees. The paste used was a resin stimulant commercial formulation composed of CEPA (2-chloroethylphosphonic acid, an ethylene-releasing compound), sulfuric acid (H_2_SO_4_), and inert components, which was applied to the trunk, after bark streak removal as previously described [14].

### 2.2. Resin Tapping

Once the treatments were randomly distributed among the trees within each site, the resin tapping operation started at biweekly intervals (BS and P treatments) [14], throughout the following two years (from spring 2009 to winter 2011). Resin collection was seasonally carried out as previously described [15], and each harvest year was named ‘crop’ since winter 2009 had passed before resin tapping started. Briefly, plastic bags were belted to trees under the wound panel to harvest resin exuded from periodically inflicted bark streaks (every 2 weeks). At the end of every season, the resin-collecting plastic bags attached to trunks were removed, rainwater was carefully drained, and the resin layer was weighed on a field digital scale (Balmak ELC-25, Santa Bárbara d’Oeste, Brazil).

### 2.3. Destructive Analysis and Carbon Quantification

**Figure 1:**
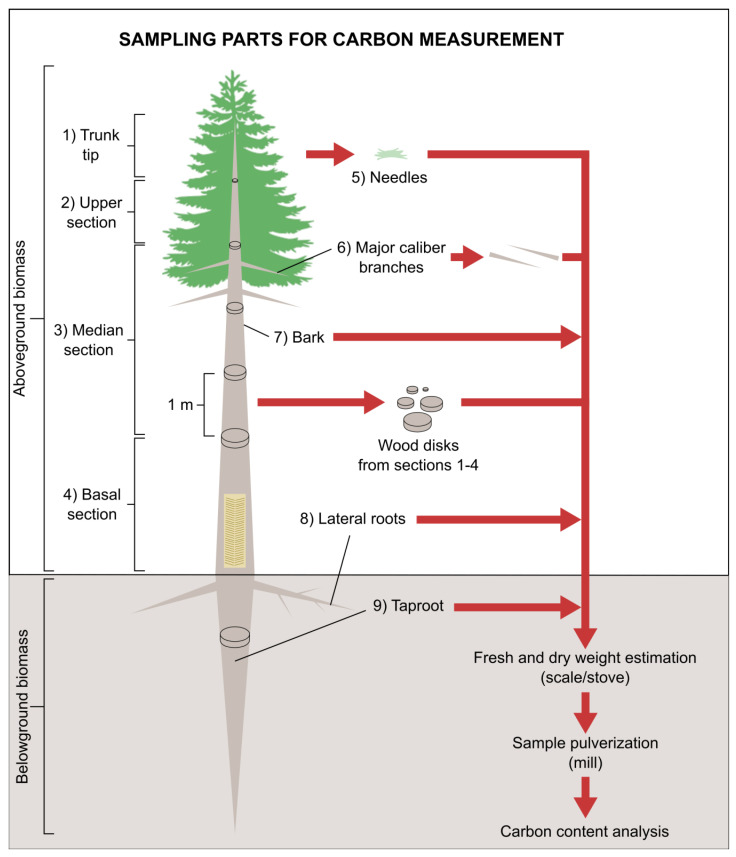
Schematic representation of plant biomass sample collection for carbon stock evaluation in slash pine trees. Five samples of every plant section indicated in the drawing were collected for every analyzed pine tree.

In November 2010, the first set of 15 trees displaying the same initial DBH range (five from each treatment) and randomly distributed in each site (see item 2.1) was felled and entirely weighed (fresh weight) in the field. Tree heights were recorded using a tape measure. All trees were dissected into their different sections as shown in Figure 1.

Aboveground biomass section boundaries were established once trees were felled. The tip section was the uppermost part with a thin and flexible stem. The other aboveground biomass sections were defined by dividing the remaining tree height by three so that equal lengths were allocated to upper, medium, and basal sections. To obtain underground biomass, the whole root system was extracted from the soil with a backhoe and washed with a pressurized water hose. Once the excess water was drained (circa 20 min), belowground biomass was sampled similarly to what was done for shoots.

Every tree section was individually subsampled as described in Figure 1, weighed, and dried in an oven at 105 °C up to constant dry weight (DW). After complete drying, the subsamples were ground in a mill to fully pulverize the plant tissues. The resulting powder was passed through a 0.15 mm sieve and subsequently evaluated for total C content through dry combustion at 900 °C on a TOC VCSH analyzer (Shimadzu, Kyoto, Japan). In November 2011, the same procedure was carried out with the 15 remaining trees of each treatment, except that only the biomass distribution of trees was measured. No direct carbon quantification was done on this occasion. Due to the inflammability, and the highly adhesive characteristic of resin, as well as for safety reasons and technical limitations of the equipment, it was not possible to directly quantify the carbon content of the resin samples. Therefore, carbon content was estimated based on the general gum rosin (C_20_H_30_O_2_) and gum turpentine (C_10_H_16_) empirical formulas [20], PubChem, https://pubchem.ncbi.nlm.nih.gov/compound/Gum-rosin, accessed 8 November 2022). The calculations considered mean values of 66% rosin, 22% turpentine, and 12% of other components. The proportions of C in the mixture (*m*/*m*) were 52.42% and 19.40%, resulting in a total of 71.81%. Hence, the estimates yielded 718 g of carbon per kg of resin.

### 2.4. Physicochemical Characterization of Soil from Pine Stands

Soil samples from 10 random spots were collected with a Dutch auger (TF 10 model, Sondaterra^®^, Piracicaba, Brazil) in each site. The materials were collected from 4 different soil depths (20 cm, 30 cm, 60 cm, and 90 cm) and were individually homogenized and the same volume of samples within each depth was combined in a single flask. Aliquots of this material were then analyzed in triplicate.

The physicochemical characterization of combined soil samples and C content assessments were performed at the Laboratory of Soils, Faculty of Agronomy, Federal University of Rio Grande do Sul (UFRGS), using conventional methods [58,59].

### 2.5. Lignin Quantification

Lignin was quantified using the acetyl bromide method [60]. Briefly, 0.3 g of dry powdered samples from four replicates randomly selected out of 14- and 24-year-old trees under three different treatments (IT, BS, and P) were homogenized in a centrifuge tube containing 7 mL of 50 mM potassium phosphate buffer and stirred vigorously. The pellet was centrifuged at 1400× *g* for 5 min and washed by successive stirring and centrifugation. The pellet was dried for 24 h at 60 °C (“protein-free cell wall fraction”). Then, a 20 mg sample was hydrolyzed in 25% acetyl bromide (*v*/*v* in glacial acetic acid) and incubated at 70 °C for 30 min for digestion. After lignin solubilization and centrifugation, absorbance was measured at 280 nm and compared to a serial concentration standard curve of alkali lignin. Data were expressed as percent lignin in the cell wall. Due to the similar age of stands at sites B and C, only the lignin content present in plant tissues from trees of sites A and B was analyzed.

### 2.6. Statistical Analyses

Initially, data were submitted for the evaluation of normal distribution (Levene test, *p* ≤ 0.05). Data sets meeting normal distribution requirements were submitted to a one-tailed t-test (comparisons involving only 2 treatments) or one-way ANOVA followed by the Tukey test. Similarly, for data sets without variance homogeneity (for 2 sample comparison, Figure S3), the Wilcoxon test was applied. In every case, *p* ≤ 0.05 was used. Tests were done using GraphPad Prisma software version 7.00 (Dotmatics, Boston, DC, USA). Resin yield was measured with 30 biological replicates. Biomass and carbon data were obtained with 5 biological replicates. Soil analyses were done in triplicate of 10 combined samples per site. Lignin data had 5 biological replicates.

## 3. Results

### 3.1. Tree Evaluation Parameters

#### 3.1.1. Tree Height

Tree density was 900 trees per hectare (ha) at sites A and C, and 600 trees per ha at site B. In the first year, on average, the highest trees were found in site C, the oldest pine plantation (22.38 ± 0.34 m) (Table 1). Statistical differences in tree height among treatments were only noticed in site B during the first year, and site C in the second year of evaluation (Figure S1a). In the first case, BS trees of 24 years were taller than those of P and IT. In the second case, P trees of 26 years were shorter than those of BS and IT.

#### 3.1.2. Tree Biomass

Not surprisingly, among the three pine plantations, total dry tree biomass was higher in sites B and C (the ones with older trees) than in site A for both years (Figure S1b). In addition, considering the plant parts separately, significant differences were only recorded for shoot biomass. In both evaluated years, shoots from sites C (26-year-old) and B (24-year-old) showed higher dry biomass than those from site A. Site A (14-year-old) average shoot dry biomass varied from 204.43 ± 9.85 kg (first year) to 213.41 ± 10.43 kg (second year) (Table 1). In the same site, tree dry root biomass was 43.40 ± 3.14 kg in the second year (Table 1). Regarding the effects of the treatments on biomass accumulation, statistical difference was only observed in the second year of evaluation in the 26-year-old site (Figure S1c). In this site, pine trees from bark streak and intact treatments exhibited total biomass of 358.42 ± 20.75 kg and 363.89 ± 10.74 kg, respectively, significantly higher than that of trees treated with a paste which had 261.39 ± 28.62 kg.

In the first year, the root-shoot biomass ratio (R:S) was 0.203 ± 0.012, 0.132 ± 0.006, and 0.154 ± 0.016 for sites A, B, and C, respectively. Site A differed from B and C which were equivalent. In the second year, these values increased slightly for all three areas, reaching 0.205 ± 0.014, 0.151 ± 0.024, and 0.160 ± 0.010 for sites A, B, and C, respectively, becoming statistically equivalent. The total wood biomass partitioning of belowground and aboveground compartments (disregarding the effect of the treatments) was similar for the three evaluated sites (Figure S2).

#### 3.1.3. Tree Diameter at Breast Height

Overall, IT trees from all evaluated sites showed the highest final DBH values (Figure S3). Since in BS and P trees, part of the bark was removed to apply the treatments, this is not surprising, which also explains the final DBH being lower than the initial one for the P trees in sites B and C, in the first and second year, respectively. No differences were found among treatments in the wood lignin content of plants from sites A and B (Figure S4).

### 3.2. Resin Yield

**Figure 2:**
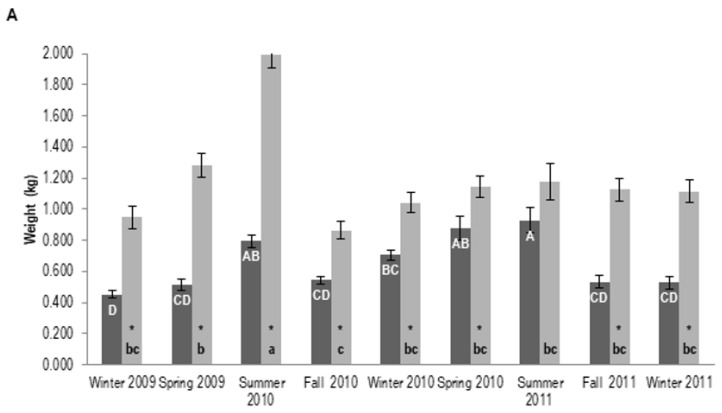

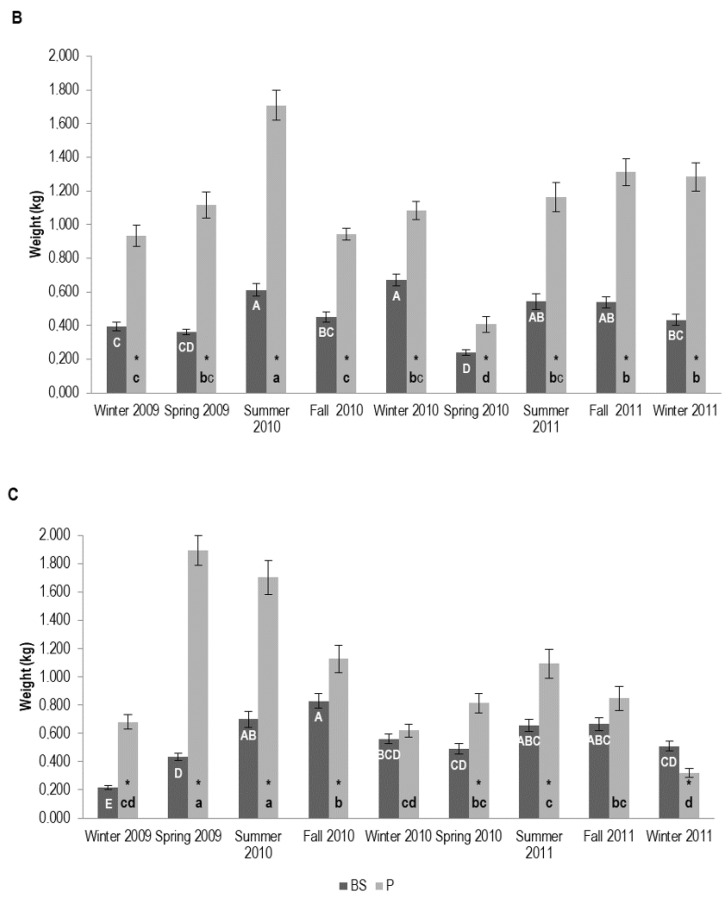
Seasonal resin production in three different slash pine plantations. (**A**) (Site A): 14-, (**B**) (Site B): 24-, (**C**) (Site C): 26-year-old stands (age at the installation of the experiments). Uppercase and lowercase letters compare resin yield within bark streak (BS) and paste (P) treatments, respectively, through different seasons from winter 2009 to winter 2011. Bars sharing a letter are not significantly different by the Tukey test (*p* ≤ 0.05). * Indicates the significant statistical difference between BS and P treatments in the same season by t-test (*p* ≤ 0.05). Note that in the southern hemisphere, the year change occurs during summer. This season commences either on December 21st or 22nd, depending on the summer solstice.

Overall, pine trees of P treatment yielded higher amounts of resin when compared to BS ones throughout the seasons and crop years evaluated (Figure 2A–C), except for site C in the winter of 2011 (Figure 2C). The overall superior induction of resin by P versus BS was also conspicuous when total resin production was considered (Table 2).

The most productive seasons for resin yield were spring and summer in the first crop year of the sites analyzed (Figure 2A–C). In contrast, in the second crop year, these seasons were not as productive (Figure 2A–C). The highest amount of chemically induced resin was found at site A in the summer of 2010 (1.997 kg per paste-treated tree) (Figure 2A). In the second crop year (from winter 2010 to winter 2011), the induced resin yield was similar throughout the seasons for site A. Conversely, the 2010 spring yield at site B was sharply lower than that recorded for all other seasons (Figure 2B).

Despite plantation age and its lower value measured for height and wood shoot biomass (Table 1), the overall highest total resin yield in the two years examined was recorded in the youngest pine plantation (site A) (Figure 2A; Table 2). This was particularly observed in the trees that did not receive paste application.

One of the main physical edaphic differences among the soil samples collected from the three study localities was the clay percentage, which was higher at site A, for all analyzed soil layers (Table S1). In addition, only site A was submitted to an intermittent flooding period.

**Figure 3:**
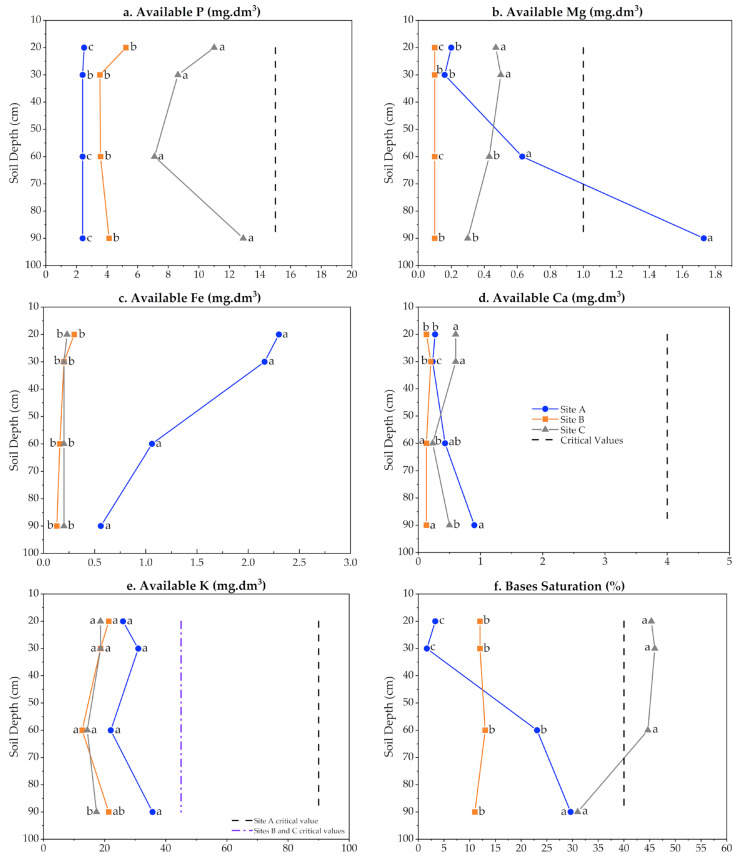
Mineral availability in soil from three different slash-pine plantations. Site A: 14-, Site B: 24-, Site C: 26-year-old slash pine plantations (age at the installation of the experiments). Each mean was calculated with ten combined samples from the same soil layer. Markers sharing a letter (within the same depth) are not significantly different by the Tukey test (*p* ≤ 0.05). Site A: blue line; Site B: orange line; Site C: gray line. Critical values are shown as dotted vertical lines (**a**,**b**,**d**,**f**) or dashed-dotted vertical lines for Sites B and C (**e**) (CQFS-RS/SC 2016—Brazilian Soil Society, https://www.siabrasil.com.br, accessed 8 November 2022). In (**c**), critical values were not defined.

Albeit none of the sites of the present study were fertilized and the nutrient levels recorded indicated mostly poor substrates, some differences in soil physicochemical properties and composition were apparent. As expected from the higher amount of clay in site A, Cation Exchange Capacity (CEC) was more elevated in this site (Table S1). The availability of Mg was significantly higher in site A starting at approximately 60 cm of soil depth (Figure 3b), whereas Fe was more available throughout the soil profile, particularly in the upper strata (Figure 3c).

Examining each site separately and considering the same plant compartment, the main differences in C percentage among treatments were observed in shoots of 14-year-old trees (Table 3). Overall, higher C values were found in the BS treatment at site A. The highest C percentage was found in needles under BS treatment (52.23 ± 0.89), followed by wood collected from the trunk basal section (51.56 ± 0.59) (Table 3). In sites A and B, the needles of trees undergoing BS showed higher C percentage values than those found in the respective aboveground bark samples (Table 3). In site A, levels of C in plant sections were the same for IT and P treated trees, except for the median section, in which the latter had a higher C percentage (Table 3). For the 24-year-old (B) stand, considering the same plant compartment, differences were only observed for needles between the IT and BS treatments. In IT trees, the C percentage was lower for needles compared to that estimated for the taproot and secondary and tertiary roots, as well as for the wood from the median trunk section (Table 3). The lowest C percentage among all sites was found at site C in the taproot sample (41.14 ± 0.87) of BS trees. No statistical differences were observed in the 26-year-old pine plantation within the BS and P treatments (Table 3).

### 3.3. Carbon Content in Plant Tissues

**Figure 4:**
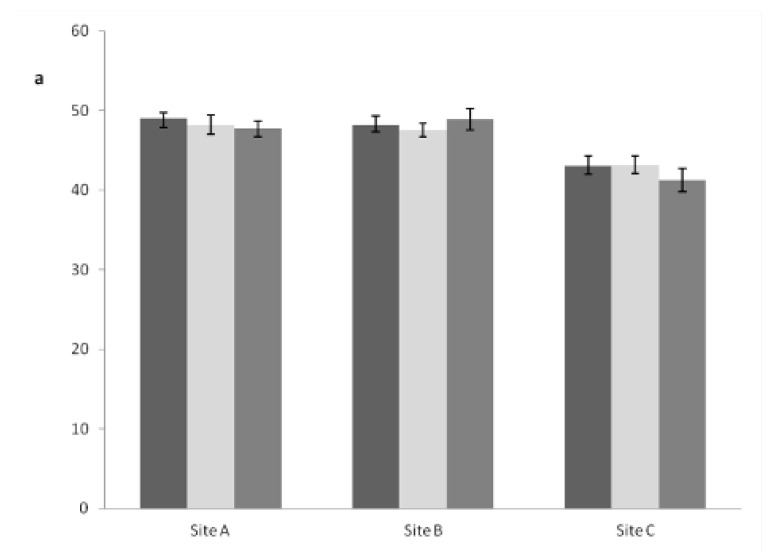

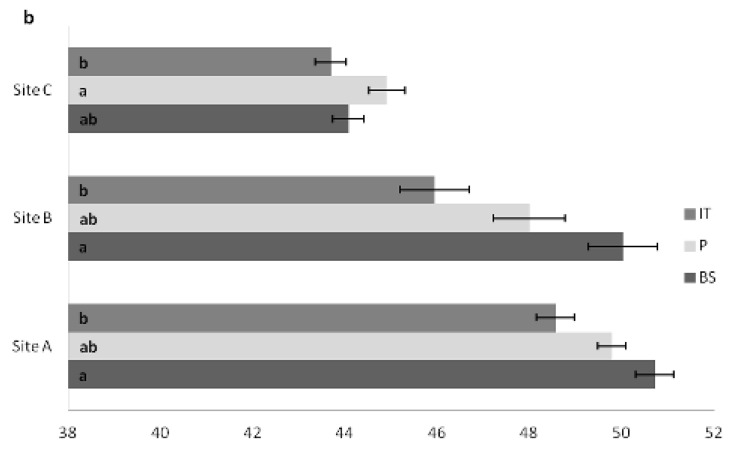
Average total carbon percentage in roots (**a**) and shoots (**b**) of slash pine trees of three different plantations (measured in the year 2010). Comparisons were valid only within the respective sites. Site A: 14-, Site B: 24-, Site C: 26-year-old plantations (age at the installation of the experiments). Treatments: IT = Intact; BS = Bark Streak; P = Paste. Each mean was calculated with 30 individual trees. Bars sharing a letter are not significantly different by the Tukey test (*p* ≤ 0.05).

The average percentages of total aboveground C content were 50%, 48%, and 44% for sites A, B, and C respectively. For belowground biomass, the total C content values were found to be approximately 48% (sites A and B), and 43% (site C) (Table 3). The total belowground biomass C percentage was not affected by the treatments in any of the sites (Figure 4a). Differences among treatments within each site were only observed for total aboveground biomass. In sites A and B, trees submitted to the BS treatment showed a higher average C percentage than trees under the IT treatment, whereas in site C, trees under P treatment had a higher C percentage compared to their IT counterparts (Figure 4b).

**Figure 5:**
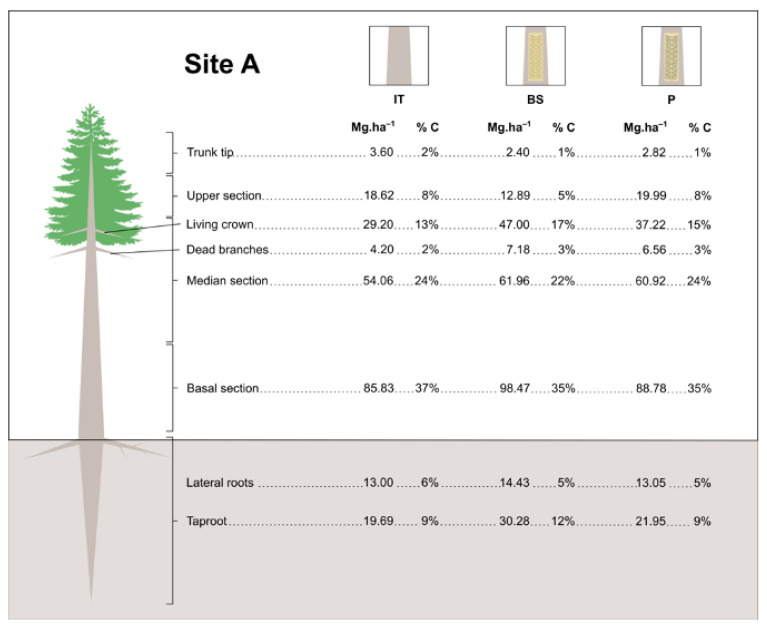

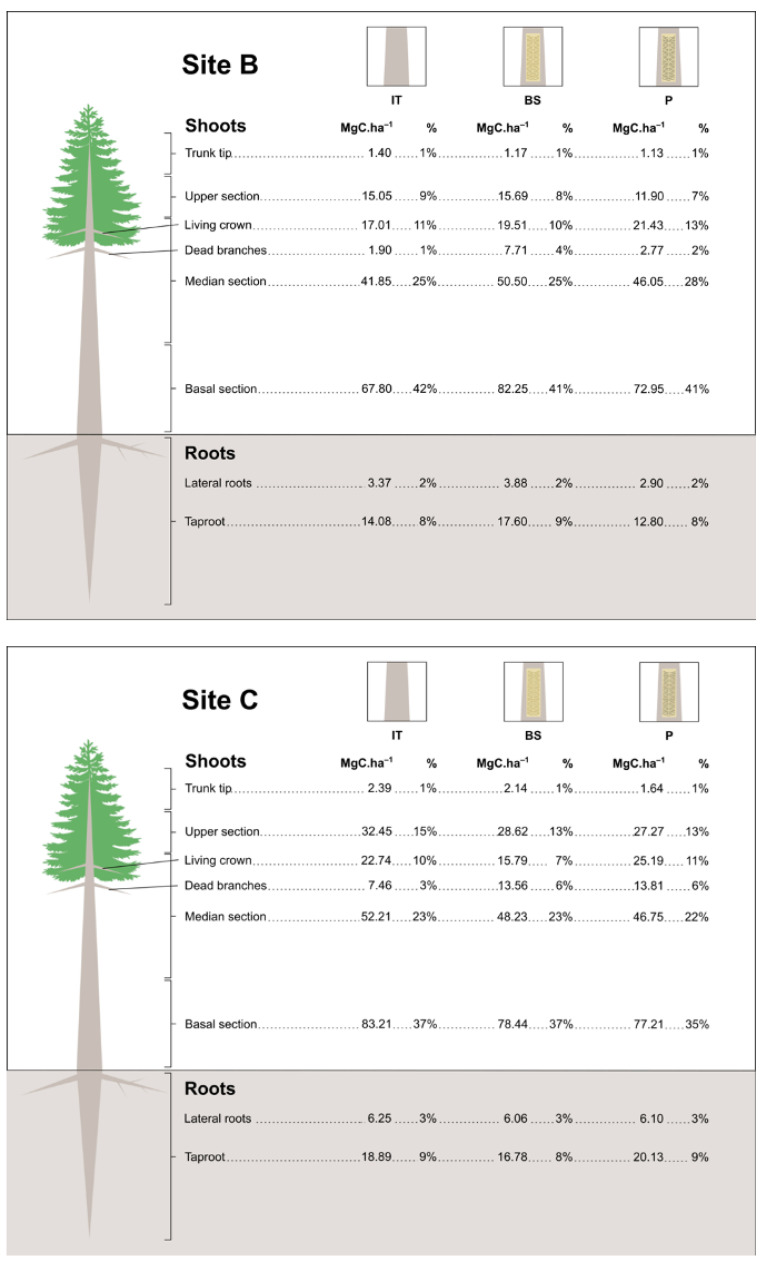
Biomass and C partitioning of slash pine trees growing at plantations of three different ages. Site A: 14-year-old, site B: 24-year-old, site C: 26-year-old (age at the installation of the experiments). Treatments: IT = Intact; BS = Bark Streak; P = Paste. The values for wood biomass (%) and carbon stock (MgC·ha^−1^) were calculated based on the biomass weight (kg) of 15 trees per treatment per pine stand (Year I). A living crown refers to live branches and leaves.

Overall, treatments had no major impact on C stocks in the biomass of trees from the different sites (Table 4). Considering the average of the three treatments per site, despite showing the lowest tree density (600 trees/ha), the highest C total stock was recorded for plantations in site B (24-year-old; 167.254 MgC·ha^−1^) in the second year of evaluation. This is consistent with the combined weight of trees growing at that site (S1a) and their total C percentage (Table 3). On the other hand, the lowest C content was found in site A (14-year-old; 123.339 MgC·ha^−1^) in the first assessed year (Figure S5). These data are compatible with the lowest weight displayed by the trees growing in site A, although their mean total C percentage was the highest (circa 50%, Table 3) of the pine stands. Analysis of C stocks in the different plant sections showed higher C stocks in stems (basal and median sections) than branches and leaves (often referred to as living crowns) (Figure 5).

Values of C sequestered by trees in sites A and C were not statistically different (Figure S5). Trees growing in site A had relatively low weight (Figure S1b). Trees in sites B and C were similar in age and mass. Despite showing 300 trees/ha less than site C, an equivalent total C stock was seen for site B.

### 3.4. Estimates of Carbon Stock in Resin Biomass

The estimated resin carbon stock was 718.1 g per kg. Therefore, considering the different site densities, as well as the annual average resin production per chemically stimulated tapped tree, the estimates of C stocks in resin biomass in the first year were approximately 3.362, 2.095, and 3.464 MgC·ha^−1^ for sites A (14-year-old), B (24-year-old), and C (26-year-old), respectively (Table 5). Given the reduced resin yield per individual in nonchemically induced trees, this treatment had lower C stocks in resin per planted area during the same year (1.660, 0.905, and 1.636 MgC·ha^−1^ for sites A, B, and C, respectively). Overall, site A was the most productive and site B the least. Similar profiles were recorded during the second year. The second year registered lower C stocks in resin biomass as expected from the diminished resin yield per tree of the different sites and treatments within the period (Table 5).

### 3.5. Soil Physicochemical Characterization and Its Carbon Content

As previously mentioned, site A had an intermittent flooding period. Sites B and C exhibited well-drained soils throughout the year.

Overall, soil samples from all tested sites displayed acidic pH values (from 4.30 ± 0.10 to 5.83 ± 0.06) (Table S1). Soil physicochemical characterization showed some heterogeneity among sites (Figure 3a–f). The main differences were observed in the concentrations of phosphorus (P) (higher at site C; Figure 3a), magnesium (Mg) (Figure 3b), and iron (Fe) (higher at site A; Figure 3c). As expected for acidic soils, very low concentrations of calcium (Ca) were found in all analyzed depths at all three sites (Figure 3d). Site A showed the highest concentration of Fe in all four evaluated depths (Figure 3c), as well as higher absolute K levels which, however, were not statistically significant in most cases. The acidic site C soil presented the highest pH values, P (Figure 3a), and Cu (Table S1) concentrations for all monitored depths. The concentration of Mg increased with depth in site A, particularly at 90 cm (Figure 3b).

Differences in cation exchange capacity (CEC) (concentration in cmol·dm^3^ and saturation percentage) and clay content were also observed among the three analyzed sites (Table S1). The presence of clay can directly affect water availability in soil layers. Regardless of the evaluated depth, the highest clay percentage and CEC concentrations were found in soil samples collected from site A (Table S1). Both the saturation percentage of CEC and the Al levels were high in site C (Table S1 and Figure 3f). Nevertheless, higher levels of bases were found only at the three more superficial layers evaluated in this site (Figure 3f).

**Figure 6:**
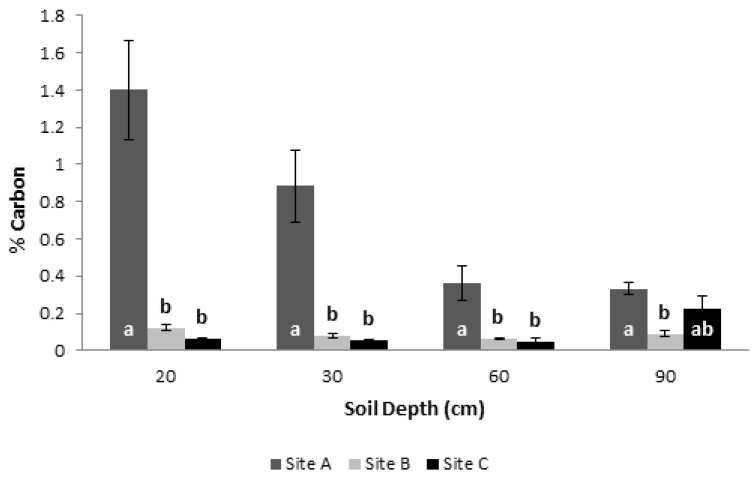
Soil carbon percentage from samples collected at different soil depths (20, 30, 60, and 90 cm), in three slash pine plantations featuring different ages. Site A: 14-, Site B: 24-, Site C: 26-year-old plantations (age at the installation of the experiments). Each mean was calculated with ten combined samples from the same soil layer. Bars sharing a letter are not significantly different by the Tukey test (*p* ≤ 0.05).

Regarding soil organic carbon (SOC) content, although significantly higher values of soil organic matter (SOM) were found at 60 and 90 cm depths in site C (Table S1), the highest available soil carbon percentage was found in site A (which assembles the youngest trees) in all the analyzed layers (Figure 6). No statistical differences in carbon percentage were found between sites B and C in all of the tested depths (Figure 6). Albeit different, the overall SOM percentage and soil carbon content were very low in all locations and depths (Table S1 and Figure 6).

## 4. Discussion

### 4.1. General Considerations

Although several studies have been carried out on carbon sequestration in native pine forests in temperate zones, there is little information available regarding the carbon stock of pines growing outside their original habitat. Even less information is available on the role of resin tapping in carbon levels and the distribution in trees. In the present work, the profile of pine carbon sequestration was determined under a subtropical climate, specifically in a coastal area. In addition, the increment in overall carbon sequestration represented by the stocked carbon in resin biomass, a copious and valuable nonwood pine product, was examined.

Carbon storage can be influenced by different factors such as climate, soil type and dynamics, physiological status of vegetation related to age [6], functional group [26], and fertilization [8,61,62], among others. Therefore, considering the different densities and ages of the three tested sites, legitimate comparisons of C content among treatments can only be made based on data acquired within the same pine stand.

This work provides a comprehensive description of carbon concentrations within the different plant compartments of pines tapped for resin production, using destructive analysis. Except for the aboveground biomass observed in the youngest analyzed site, the total carbon concentration percentage present above- and belowground was lower than the 50% predicted in the pertinent literature. On the other hand, intratree differences were seen at least in one treatment of the three evaluated sites. Comparing the plant sections, the only predominantly observed allocation pattern was lower carbon concentrations in roots than in shoots in all three analyzed sites.

### 4.2. Biomass Aspects

Considering equivalent ages, low values of biomass were found for pine species in temperate zones when compared to those recorded in the present work (in the 14-year-old plantation, circa 213 and 43 kg per tree, for shoot and root, respectively). A 15-year-old native forest of *Pinus strobus* L. (eastern white pine) displayed mean above- and belowground dry biomass of 54 and 13 kg per tree, respectively [42]. In a 17-year-old native slash pine plantation, the biomass allocations for stems, branches, and needles were 75.6, 5.7, and 4.2 Mg·ha^−1^, respectively [27], roughly equivalent to 51.3 kg of shoots per tree, considering the plantation spacing. As expected, higher values of dry tree biomass than the ones found here are registered only in much older pine forests from temperate zones. For example, a 65-year-old eastern white pine stand had a dry biomass of 529 and 99 kg per tree for above and belowground parts, respectively [42]. In the present study, the highest total biomass was recorded in sites B and C in both assessed years. This is not in agreement with the prediction for low-density tree-stand biomass, considering that site C displayed 300 additional trees per hectare in relation to site B. On the other hand, site C featured the highest tree average height in our study (Table 1).

In agreement with their higher total average shoot and root biomass production, sites B and C showed similar carbon stock values in the two consecutive years (Figure S5), despite showing different carbon average percentages. Among the three tested sites, trees at site A invested the most in height (more than 1.0 m·tree^−1^·year^−1^) as well as in resin production (more than 4.8 kg·tree^−1^·year^−1^). Equivalent investment in wood biomass was seen for sites B and A (4.73% and 4.33%, respectively) in the second year. On the other hand, site C exhibited decreased biomass in the second year—by 6.73%. Work on Scots pines resin responses to artificially inoculated *Ophiostoma brunnneo-ciliatum* led to the proposal that at young ages pines share photosynthates from the current photosynthesis process between wood biomass acquisition and induced-resin biosynthesis, whereas mature trees mainly rely on stored carbohydrates for the latter [63]. This agrees with the results found in the current study for sites A and B which showed reduced oleoresin production in the second year compared to the first one and invested the most in wood biomass production compared to site C. The same is not valid for site C, with minimum biomass investment in both oleoresin and wood. In fact, it does not seem to show the typical growth-differentiation balance hypothesis profile regarding resin biosynthesis, at least for the second year of the experiment [64].

### 4.3. Carbon Ratio

Carbon percentage values observed for sites A and B were consistent with a destructive carbon analysis performed in maritime pine plantations ranging from 1 to 47-year-old plants. In those areas, the carbon content average was 48.1% and 50.5% for root and shoot biomass, respectively [65]. For plants of this same species growing in a 50-year-old native pine forest, mean carbon concentrations of 53.6% in shoots and 51.7% in roots were recorded [32]. Studies with *Pinus* spp. plantations in southern Brazil (mainly loblolly and slash pines not tapped for resin production) used different estimated average carbon contents per tree compartment, including needles (41%), branches (45%), roots (44%), and trunks (45%) [66]. These values were generally lower than those of the present study (Table 3).

The higher carbon stocks observed in stems (basal and median sections) than in branches and leaves (often referred to as living crowns) agree with the findings for loblolly pine [43]. Overall, carbon stocks recorded in all three sites (Table 4) were higher than values reported for other pine stands, even if superior tree densities are considered. For instance, lower carbon storage was found in an exotic 21-year-old slash pine plantation with superior site density (1,439 trees ha^−1^) in a subtropical climate (116.77 ± 7.49 MgC·ha^−1^) [67]. Similar results were observed for a 15-year-old jack pine (*Pinus banksiana* Lamb.) stand with a density of 2,600 trees·ha^−1^ and carbon stock of 103 MgC·ha^−1^. In the same study, 24- and 26-year-old *Pinus resinosa* Ait. (red pine) stands featuring 1,360 and 1,800 trees·ha^−1^, stored 106.13 and 152.60 MgC·ha^−1^, respectively [44]. In a native 50-year-old maritime pine stand with a density of 223 trees·ha^−1^, carbon content was 74 MgC·ha^−1^ [32]. Studies on the development of allometric equations for *Pinus* spp. (growing on plantations in southern Brazil not tapped for resin production) also found lower carbon stocks for 15-year-old pine plantations, roughly 114 MgC·ha^−1^ [68] and 102 MgC·ha^−1^ [66]. An investigation of loblolly pine in southern Brazil reported carbon stocks in trunk biomass of 41.8, 91.4, and 91.9 MgC·ha^−1^ in 14-, 25- and 26-year-old exotic stands, respectively [69].

### 4.4. Water Availability

Usually, the most productive seasons for stimulated resin yields in southern Brazil are spring and summer [11,15], which was the case observed in the first year of the present study, but not in the second one. This may be explained by differences in rainfall. The average seasonal rainfall in sites A and B was 29% higher in the first year compared to the second one. A similar pattern was observed in site C that showed a seasonal average rainfall of 367.8 mm in the first year (35% higher than the one registered for the second year) (INMET, 2022, https://tempo.inmet.gov.br/TabelaEstacoes/A001, accessed on 3 December 2022).

Water availability seems to be a crucial factor affecting pine resin biosynthesis [11]. Both high water availability and moderate water stress have been shown to increase resin yields in different pines and other Pinaceae species. Under moderate water stress, sufficient to limit plant growth, constitutive resin flow was enhanced in full-grown *Pinus taeda* L. (loblolly pine) trees. On the other hand, inducible resin exudation in this species was higher during the season of greatest growth, in the fastest-growing trees [70]. A similar constitutive response was observed in *Pinus sylvestris* L. (Scots pine). In this species, changes in the terpenoid profile and concentration were only detected when plants experienced moderate to severe water stress, after photosynthesis limitation due to stomatal closure [71]. In Scots pine, a suitable water supply in dry sites indirectly affected resin biosynthesis by means of radial growth promotion [72]. In *Abies grandis* (Douglas ex D. Don) Lindley, a species belonging to the Pinaceae, water and light stress acted as negative modulators of constitutive-monoterpene cyclase activity in both saplings and adult trees [73].

### 4.5. Edaphic Factors

The presence of more clay and intermittent flooding in site A may have interfered with water availability at the rhizosphere, potentially stimulating resin biosynthesis in the shoots. Hypoxia conditions in flooded roots may induce the accumulation of ethylene precursors which move to the shoots and subsequently promote ethylene production, thereby stimulating resin biosynthesis and flow [74,75]. Thus, high water availability at this site might have promoted resin yield.

Most studies have reported negative or no effects of fertilization on resin flow [76]. In 6- and 12-year-old stands of loblolly pine trees, constitutive resin flow was increased by fertilization. However, only the younger trees were able to keep the resin flow after wounding and fungal inoculation treatments [77]. Terpene chemical profiles and emissions could also be altered by fertilization in 50-year-old Scots pine trees, and the profile of resin acids from sapwood was more responsive to nitrogen (N) treatment than monoterpenes from heartwood [78]. In Scots pine growing at polluted sites in Finland, fertilizer treatments containing N decreased resin flow in treated plants [79]. Eleven-year-old plants of loblolly pine that were N-, P-, K-, Mg-, Ca-, and B-fertilized yielded 30 to 100% less resin compared to untreated trees [80].

The higher CEC in site A (higher in clay relative to the other sites) may have contributed to the nutrient presence in the soil, as well as acting as a buffer against excessive acidification. The higher availability of Mg and Fe in the same site may also have modulated resin yield. Aside from being essential for numerous cellular functions that support growth, Mg and Fe are required for the activity of one or more classes of pine terpene synthases and their use as resin stimulant paste adjuvants has improved yields in slash pine [16]. Therefore, in addition to the impacts of DBH and water availability on the resin yield of site A trees, the higher soil availability of these two cations might have also contributed to resin biosynthesis in the 14-year-old plantation.

Of all elements assessed (Figure 3a–f and Table S1), potassium (K), copper (Cu), manganese (Mn), and iron (Fe) are known to be key cofactors of terpenoid biosynthetic enzymes involved in resin biosynthesis that can impact yield [16,18]. Soil mineral availability depends on different factors such as pH, mineral soil-plant mobility, and mineral complexation with soil particles or other chemical elements. Mycorrhizal associations with pines are also relevant, particularly for P, but also for N and K acquisition in poor soils [81,82].

Soil acidification promotes the formation of Al toxic species, which reduces the mineral availability in soils, including P. Regarding fertilization, the growth response in loblolly pine (an Al-sensitive species) was more correlated to extractable Al indices than to N or P availability [83]. Root injury preceded by mycorrhizal activity inhibition is a common indicator of Al toxicity. The uptake and distribution of Mg, Fe, and Mn in shoot and root tissues of *Pinus massoniana* Lamb. (masson pine) were altered by Al solution treatment. The typical root growth inhibition, related to the mitotic imbalance caused by chromosome aberrations, was also seen in masson pine seedlings because of Al accumulation in roots [84].

The negative effect of Al on P availability has been described for various forest stands and it may partly explain the low P concentration in site A soil. In maritime pine, depletion of soil P was observed to be more limiting for growth than for leaf terpene biosynthesis [85]. Lime application on a 20-year-old exotic plantation of slash pine in China was more effective to improve resin yields than NPK fertilization [86]. However, liming might be a counterproductive practice in terms of the maintenance of soil carbon stocks since it represents a direct source of CO_2_ emissions to the atmosphere [1].

Soil acidic conditions are often not favorable to pine species cultivation. As previously noted, the overall highest exchangeable and available concentration of Al was found at site A, along with the lowest mean pH value in all soil depths. An overview of soil features of the experimental sites in the present study points to site A as the most stressful one, substrate-wise. Despite this condition, the site also yielded the highest total resin average per tree in both years of evaluation. As previously pointed out this profile may also have been affected by the local intermittent flooding events.

Along with carbon flows in forest vegetation, soil organic carbon (SOC) dynamics can be variable and dependent on different factors such as density, management practices, site conditions, and preceding use of the land [53]. The SOC values recorded (ranging from 30 to 115 Mg·ha^−1^) were relatively low compared to those found in soil samples (until 100 cm depth) from a five-year-old loblolly pine plantation (227.8 Mg·ha^−1^) not tapped for resin production, growing in a physiographic region in southern Brazil named “Campos de Cima da Serra” [87], which is located approximately 1240 m above sea level. In the current work, however, all locations were typical coastal sandy soils without recent prior plant cover, and poor in organic matter.

In 22 years-old plantations of masson pine (a native species) and slash pine (an alien species) grown in subtropical China, a similar contribution of both species to SOC was recorded [88]. Data from more than 400 sites in Poland showed that soil from pine stands contained less stored carbon than that of other coniferous species, like fir (*Abies* spp.) and spruce (*Picea* spp.). Soil stored carbon was also higher in deciduous tree stands such as beech (*Fagus* spp.) and oak (*Quercus* spp.) compared to pine areas. Moreover, the lowest carbon stocks were found in the low pH range (4.5–5.5) [89]. Lower SOC values were also recorded in forests of *Pinus koraiensis* Siebold and Zucc. (Korean pine) compared to birch (*Betula platyphylla* Sukaczev) and dahurian larch (*Larix gmelinii* (Rupr.) Rupr.) stands [90]. Clay may also affect the soil’s carbon pool.

In temperate zones, the climate found in elevated areas, characterized by higher precipitation and lower temperature, is an important factor affecting the carbon stock in forest soils [89]. In China, it has been shown that the soil carbon stock increases with altitude in secondary coniferous forests such as *Larix principis-rupprechtii* Mayr, *Picea meyerii* Rehder & E.H. Wilson and *Pinus tabulaeformis* Carr. [51]. In the present study, all assessed sites were located at sea level, and close to a coastal region; hence, lower values in carbon estimates are expected compared to other landscapes.

### 4.6. Resin Yield

The fact that the youngest plantation (site A) yielded the overall highest resin in the two years monitored may be partly explained by the larger mean initial DBH found at this site [14] and possibly by higher numbers of radial resin ducts present in the wound panel [91]. Particularly for slash pine, the number and size of resin ducts (and therefore resin biosynthesis) are higher and usually more active in young trees. The number of ducts can decrease with age up to 20 years old, whereas resin canal size may decrease at least up to 30 years of age in trees [92,93]. Resin duct area and size have been shown to strongly correlate with resin yield in slash pines of three different locations in China [94]. Moreover, it is well known that pine resin biosynthesis responds to a multitude of intrinsic and environmental factors, such as plant genetics, age, water, temperature, and mineral nutrient availability, among others [13,18,74,76,78] as discussed above in Section 4.3 and 4.4. As expected, sulfuric acid plus ethylene stimulant paste application increased significantly the production of resin. These adjuvants act by triggering and intensifying defense responses to wounding, which are mostly related to the exudation of this complex mixture of terpenes. Overall, the most productive resin yield seasons were the warmer ones, whereas winter yields were often reduced, in agreement with the usual profile [9].

Pine resin is made up of a volatile fraction (turpentine), majorly composed of monoterpenes and a few sesquiterpenes, and a nonvolatile fraction formed by diterpenic acids (rosin) [21]. The crude resin composition, in terms of turpentine-rosin proportion, was variable and site- and species-dependent. For instance, the analysis of 22 Chinese pine species from subgenus *strobus* showed that diterpenes comprise 59.5 to 80.9% of the produced resin [95]. In European black pine (*Pinus nigra* spp. *laricio* J.F. Arnold) this resin fraction is between 46 and 66% [96]. In maritime pine, more than 70% of the crude resin is made up of diterpene acids [97]. Turpentine yields in natural populations of *Pinus merkusii* Jungh. and de Vriese are in the range of 28.5 to 32.8% (*v*/*w*) [98]. In slash pine, turpentine represents 22 to 25% of the resin weight [20,99], and is mostly composed of α- and β-pinenes [16,20].

Pine resin subproducts have several applications in the chemical industry. For instance, turpentine components are usually employed in the production of solvents or cleaning agents for paintings and varnishes, pine oils [21,57], insecticides, and essential oils of flavorings and fragrances [100]. Rosin constituents, in turn, are used as feedstock for more long-lasting products such as adhesives, synthetic rubber, coatings [100], waterproof materials, inks, paper sizing, and rubber emulsifiers [21]. Thus, regarding residence time [101], besides enhancing carbon sequestration in pine plantations, resin utilization also contributes to carbon fixation and permanency, mostly due to the long lifetime of its nonvolatile fraction derivatives.

### 4.7. Tree Development

Considering the highest density and carbon percentage observed in site A, higher carbon stocks were expected in the younger stand versus similarly spaced trees of the oldest site, C. The observed absence of difference in sequestered carbon was probably due to the low weight of the trees growing at site A. Sites B and C had similar ages and trees with comparable average weights. Although site B was less dense than site C, carbon stocks were equivalent. This discrepancy in carbon stocks related to the plant age found in the present study may be partly explained by the management status of the pine stands. Sites A (14-year-old) and B (24-year-old) were thinned at the ages of 10 and 15 years old, respectively, whereas site C (26-year-old) was kept undisturbed since stand establishment. Moreover, local climate, site density, distinct soil traits, and the impact of resin tapping activity previously performed in the tested areas (during two consecutive years), should also be considered potential factors influencing carbon stock capacity.

Age influence on carbon storage in our study was comparable to that found in forests of *Pinus ponderosa* Douglas ex C. Lawson (ponderosa pine) at different developmental stages. In ponderosa pine stands, total carbon stocks were higher in the older area (never logged) when compared to the younger one (previously clearcut) [26]. Similar results were observed in red pine stands. Carbon stocks increased with plant age in thinned stands. However, such an increase was only observed until the middle of the observed chronosequence in unmanaged stands [5]. Indeed, younger stands are expected to sequester larger carbon amounts compared to older ones, since their larger carbon uptake is associated with active growth. Older forests, in turn, generally display a limited growth rate and higher carbon stocks [29].

An average increase of 5.53% and 4.73% in wood biomass for sites A and B, respectively, and a reduction of 6.73% in site C, were observed within the time monitored (Figure S1b). On the other hand, plants of all three sites reduced resin exudation from the first to the second year (crop), especially in the chemically induced pine trees (Table 2). Considering the average amount of resin produced by chemically stimulated and nonchemically stimulated trees within the same site in the two assessed years, the youngest plantation (14-year-old) produced the highest total resin biomass in both crops, while the 24-year-old site produced the lowest (Table 2). The greater values of resin observed in the first year (crop) might be explained by the constitutive storage of this biomass in tree trunks before resin tapping operations started. In addition, rainfall varied through the evaluated years.

Allometric equations are very useful to predict increases in the biomass of pine plantations, however, they are accurate only if developed for the site- and species-specific traits [30]. For example, data collection of 77 Scots pine stands aged from 3 to 20 years showed that tree stand biomass increases with tree height and volume as well as with tree age. On the other hand, tree biomass decreases with higher stand density in the evaluated chronosequence [37].

In both evaluated years, the highest total biomass was seen in sites B and C. This fact is not in agreement with the prediction for low-density tree stand biomass, considering that site C had 300 additional trees per hectare in relation to site B. On the other hand, site C featured the highest tree average height in our study (Table 1).

Despite its lower tree density, site B did not show a higher *R:S* biomass ratio. In *Pinus pinaster* (Ait.) (maritime pine) growing in southwestern Australia, the *R:S* was higher in sites featuring open-spaced trees rather than in those with close-spaced trees of the same size [65]. In agreement with the present study for pines growing in the same geographic area (sites A and B), an overall decrease in *R:S* with increasing age was reported in eastern white pine stands [42].

### 4.8. Silviculture, Landscape Management, and Policy

It is well established that different silvicultural practices [48,102] and plant ages [43,45] can influence carbon sequestration in pine stands. The date and intensity of thinning can also impact allometric relationships and carbon intake in pine stands [49,87]. It was shown that intensive management (fertilization and/or understory elimination) can increase carbon sequestration in 17-year-old slash pine plantations growing in sandy flatwoods soils [27]. In postfire regenerated forests of *Pinus halepensis* Mill. (Aleppo pine), early thinning increased the productivity of pine saplings. On the other hand, the total quantity of carbon sequestration and partitioning decreased following intensive thinning [103], and strong early thinning in preburned sites of maritime pine negatively affected the carbon biomass of saplings [104]. A similar result was observed in even aged pure stands of maritime pine and radiata pine (*Pinus radiata* D. Don), where lower thinning intensity and higher rotation age increased the aboveground biomass and carbon pools [48]. A comparison of carbon stocks between two pine species stands under different managements also showed that thinning reduced carbon sequestration. Similar carbon sequestration values were observed between a thinned 75-year rotation of *Pinus palustris* Mill. (longleaf pine) and unthinned 25-year-old rotation slash pine [36].

As mentioned above, *Pinus* is an exotic genus in Brazilian territory. Due to its invasiveness potential, the State Environmental Authority (SEMA) Normative Instruction n° 14 of 10 December 2014 (www.legisweb.com.br/legislacao/?id=278555, accessed 8 November 2022) established that pine plantations in southern Brazil must be restricted to areas previously occupied by species of this genus. As a result, the search for alternative commercial activities has increased to ensure the optimization of land use before tree logging, in addition to postponing the time-demanding regeneration process of the pine stands. On the other hand, unlike timber extraction, pine resin represents a short-term abundant, sustainable, and renewable carbon biomass source. Therefore, in southern Brazil, resin tapping operations have recently been intensified as a profitable activity that indirectly contributes to the local mitigation of greenhouse gas effects. Overall, despite the not unexpectedly low soil carbon stock, plant biomass total carbon stock was higher for all the tree analyzed ages in comparison to the values obtained by both destructive analyses and allometric predictions recorded in the literature, considering the same or other pine species with similar ages. Annual slash pine resin production in Brazil and the C estimates herein described for this nonwood product indicate relevant carbon sequestration increments per year in tapped pine plantations. In addition, individual pine trees can provide resin for several years prior to felling, which promotes further atmospheric carbon removal and storage.

The present study provided valuable primary results regarding carbon capture and sequestration in *Pinus* plantations subjected to the influence of soil types, ages, and management practices (resin collection and chemically stimulated resin collection). Future studies can draw on these results to analyze the bioeconomic impacts of *Pinus* plantations concerning their consequences for carbon credit trading. Comparative analysis of total carbon sequestered annually per hectare may be appropriate for certification and auditing of carbon credits and the consequent decision-making by producers regarding the adoption of one or another system of forest stand management considering the highest possible additional returns derived from the marketing of carbon credits. It is hoped that this novel information on carbon stocks of exotic slash pine plantations tapped for resin will provide a framework to value the contribution of the resin industry regarding carbon credits, as well as represent an additional tool to guide decision-making in forestry policies.

## 5. Conclusions

The biomass of coastal slash pine plantations in subtropical climates is relatively high compared to that of related species of comparable age in temperate zones. This profile is seen despite limited soil fertility, variations in tree age, water availability, and site, highlighting the environmental resilience and plasticity of this forest species. Although all tree fractions contribute to carbon content, most carbon is associated with shoots, particularly trunks, an aspect to consider in genetic selection programs aimed at carbon sink activity. Resin yield constitutes a relevant component of carbon allocation and retention, notably in paste-stimulated resinosis. The inclusion of resin extraction in stand carbon credit computation is recommended, especially considering its continuous exploration over several years and the significant carbon residence time in many of its multiple derivatives.

## Figures and Tables

**Table 1:** Shoot and root growth parameters in each one of the three pine stands were measured in two different years.

Plantation Age (Years)	(Year I) Plant Shoot Height (m)	(Year II) Plant Shoot Height (m)	(Year I) Root DW (kg)	(Year II) Root DW (kg)	(Year I) Shoot DW (kg)	(Year II) Shoot DW (kg)	DBH i (cm)	DBH f_A_ (cm)	DBH f_B_ (cm)
A	17.20 ± 0.24 ^c^	18.28 ± 0.25 ^c^	41.25 ± 2.98 ^a^	43.40 ± 3.14 ^a^	204.43 ± 9.85 ^b^	213.41 ± 10.43 ^b^	23.48 ± 0.12 ^a^	24.19 ± 0.39 ^a^	23.58 ± 0.49 ^a^
B	21.31 ± 0.24 ^b^	21.57 ± 0.11 ^b^	37.34 ± 1.77 ^a^	44.01 ± 6.70 ^a^	285.54 ± 8.84 ^a^	294.89 ± 11.56 ^a^	22.77 ± 0.09 ^b^	23.24 ± 0.29 ^ab^	23.84 ± 0.41 ^a^
C	22.38 ± 0.34 ^a^	22.91 ± 0.30 ^a^	45.96 ± 4.44 ^a^	44.62 ± 2.86 ^a^	304.03 ± 15.22 ^a^	283.20 ± 17.81 ^a^	22.54 ± 0.10 ^b^	22.51 ± 0.40 ^b^	23.37 ± 0.45 ^a^

Year indicates the time when pine trees were harvested Year I: 2010; Year II: 2011. DBHi: initial diameter at breast height; measured in June 2009 at the beginning of the experiments; DBHf: final DBH; measured at the time when trees were harvested: 2010 _(fA)_ and 2011 _(fB)_. 14-year-old (Site A); 24-year-old (Site B); 26-year-old (Site C). Columns sharing a letter (within the same parameter) are not significantly different by the Tukey test (*p* ≤ 0.05).

**Table 2:** Average resin production per tree in pine plantations of three different ages.

Sites	Crop I (Spring 2009 to Winter 2010)		Crop II (Spring 2010 to Winter 2011)	
	BS (kg)	P (kg)	BS (kg)	P (kg)
Site A	2.562 ± 0.01 ^a^	5.188 ± 0.24 ^a,^*	2.867 ± 0.14 ^a^	4.426 ± 0.21 ^a,^*
Site B	2.095 ± 0.10 ^b^	4.850 ± 0.22 ^a,^*	1.737 ± 0.10 ^c^	4.181 ± 0.25 ^a,^*
Site C	2.524 ± 0.13 ^a^	5.346 ± 0.34 ^a,^*	2.241 ± 0.13 ^b^	3.098 ± 0.26 ^b,^*

Site A: 14-year-old; Site B: 24-year-old; Site C: 26-year-old (age at the installation of the experiments). CTRL: (control) bark streak treatment; Crop I: resin biomass seasonally collected from spring 2009 to winter 2010; Crop II: resin biomass seasonally collected from spring 2010 to winter 2011 (the resin exudated in winter 2009 is not considered in these values). BS = Bark Streak, P = Paste treatments. Columns sharing a letter (in the same treatment) are not significantly different by the Tukey test (*p* ≤ 0.05). * Indicates the significant statistical difference between treatments in the same crop by t-test (*p* ≤ 0.05).

**Table 3:** Carbon percentage in slash pine samples.

a. SiteA	IT	BS	P
Trunk Tip	48.15 ± 0.56 ^bA^	51.44 ± 0.88 ^aAB^	50.29 ± 0.38 ^abA^
Upper Section	49.15 ± 0.68 ^bA^	51.35 ± 0.66 ^aAB^	49.73 ± 0.29 ^abA^
Median Section	49.16 ± 0.83 ^bA^	51.34 ± 0.16 ^aAB^	51.03 ± 0.39 ^aA^
Basal Section	49.82 ± 0.76 ^aA^	51.56 ± 0.59 ^aAB^	50.70 ± 0.30 ^aA^
Branches	49.23 ± 0.74 ^bA^	51.05 ± 0.72 ^aAB^	50.47 ± 0.34 ^abA^
Needles	50.22 ± 0.88 ^aA^	52.23 ± 0.89 ^aA^	50.00 ± 1.24 ^aA^
Taproot	47.55 ± 1.81 ^aA^	48.76 ± 1.13 ^aAB^	47.15 ± 2.14 ^aA^
Lateral Roots	49.27 ± 1.16 ^aA^	49.95 ± 1.22 ^aAB^	51.03 ± 1.09 ^aA^
Aboveground Bark	44.95 ± 0.94 ^aA^	47.36 ± 1.18 ^aB^	47.22 ± 0.68 ^aA^
b. Site B	IT	BS	P
Trunk Tip	44.69 ± 1.46 ^aAB^	49.66 ± 2.08 ^aAB^	46.05 ± 0.49 ^aA^
Upper Section	46.45 ± 1.13 ^aAB^	51.12 ± 0.21 ^aAB^	48.05 ± 1.78 ^aA^
Median Section	49.75 ± 2.04 ^aA^	51.30 ± 0.43 ^aAB^	47.76 ± 1.75 ^aA^
Basal Section	46.58 ± 2.31 ^aAB^	51.44 ± 0.55 ^aAB^	51.19 ± 1.97 ^aA^
Branches	46.38 ± 1.31 ^aAB^	49.835 ± 1.98 ^aAB^	47.91 ± 2.56 ^aA^
Needles	41.81 ± 2.29 ^bB^	52.57 ± 0.41 ^aA^	49.58 ± 3.14^abA^
Taproot	49.63 ± 1.0 ^aA^	48.04 ± 2.20 ^aAB^	48.26 ± 2.71 ^aA^
Lateral Roots	49.71 ± 1.55 ^aA^	49.80 ± 0.87 ^aAB^	48.31 ± 1.31 ^aA^
Aboveground Bark	45.77 ± 0.29 ^aAB^	46.18 ± 1.14 ^aB^	45.16 ± 0.52 ^aA^
c. Site C	IT	BS	P
Trunk Tip	43.21 ± 0.79 ^aAB^	44.25 ± 0.85 ^aA^	43.59 ± 0.35 ^aA^
Upper Section	42.13 ± 0.82 ^aAB^	44.43 ± 1.00 ^aA^	44.59 ± 0.47 ^aA^
Median Section	44.92 ± 0.32 ^aA^	43.37 ± 1.16 ^aA^	45.09 ± 1.07 ^aA^
Basal Section	44.27 ± 0.58 ^aA^	43.65 ± 0.65^aB^	46.15 ± 0.72 ^aA^
Branches	44.12 ± 0.80 ^aA^	44.21 ± 0.67 ^aA^	45.54 ± 0.85 ^aA^
Needles	43.52 ± 1.32 ^aAB^	43.77 ± 1.13 ^aA^	44.94 ± 1.53 ^aA^
Taproot	42.83 ± 1.28 ^aAB^	41.14 ± 0.87 ^aA^	44.53 ± 1.38 ^aA^
Lateral Roots	42.55 ± 0.27 ^aAB^	44.93 ± 1.81 ^aA^	41.84 ± 0.44 ^aA^
Aboveground Bark	44.00 ± 1.76 ^aA^	44.64 ± 0.18 ^aA^	43.54 ± 1.58 ^aA^
d. Site	Age (years)	Total Carbon %	
A	14	49.63 ± 0.32 ^A^	
B	24	48.26 ± 0.47 ^B^	
C	26	43.92 ± 0.22 ^C^	

Lateral Roots = secondary and tertiary roots. Rows = comparison among treatments (lowercase letters); Columns = comparison among plant compartments in the same treatment (a–c), or total carbon percentage (above+ belowground biomass) among sites (d) (uppercase letters). Site A = 14-year-old; Site B = 24-year-old; Site C = 26-year-old (age at the installation of the experiments). Treatments: IT = Intact; BS = Bark Streak; P = Paste. Rows or columns sharing a letter are not significantly different by the Tukey test (*p* ≤ 0.05).

**Table 4:** Aboveground, belowground, and total carbon stock in biomass of slash pine plantations of three different ages under different tapping treatments.

	Site Age (Years)	Site Tree Density (Trees Per ha)	Treatment	Shoots	Roots	Total Carbon stock (MgC.ha^−1^)
Year I	14	900	BS	115.601 ± 5.76 ^aABC^	24.348 ± 3.14 ^aA^	139.949 ± 7.20 ^aAB^
			P	91.304 ± 7.66 ^aC^	16.799 ± 1.66 ^aA^	108.103 ± 9.19 ^aB^
			IT	102.821 ± 9.97 ^aBC^	19.145 ± 1.05 ^aA^	121.966 ± 10.44 ^aAB^
	24	600	BS	145.795 ± 8.36 ^aA^	19.863 ± 1.27 ^aA^	165.658 ± 8.85 ^aA^
			P	126.597 ± 8.43 ^aABC^	17.549 ± 2.61 ^aA^	144.146 ± 8.20 ^aAB^
			IT	119.821 ± 2.39 ^aABC^	16.530 ± 2.16 ^aA^	136.351 ± 3.19 ^aAB^
	26	900	BS	138.277 ± 16.18 ^aAB^	18.170 ± 2.99 ^aA^	156.447 ± 21.0 ^aA^
			P	129.644 ± 7.85 ^aABC^	19.582 ± 5.70 ^aA^	149.226 ± 9.79 ^aAB^
			IT	136.357 ± 8.76 ^aAB^	20.900 ± 3.46 ^aA^	157.257 ± 9.11 ^aA^
Year II	14	885	BS	100.804 ± 7.61 ^aC^	21.183 ± 3.78 ^aA^	121.987 ± 9.22 ^aB^
			P	105.193 ± 6.53 ^aBC^	19.344 ± 2.26 ^aA^	124.537 ± 8.73 ^aAB^
			IT	117.271 ± 11.27 ^aABC^	22.630 ± 2.11 ^aA^	139.901 ± 12.37 ^aAB^
	24	585	BS	151.588 ± 11.88 ^aA^	21.189 ± 6.85 ^aA^	172.777 ± 9.30 ^aA^
			P	139.260 ± 3.22 ^aABC^	16.587 ± 6.51 ^aA^	155.847 ± 8.58 ^aAB^
			IT	146.323 ± 12.77 ^aAB^	26.816 ± 3.78 ^aA^	173.139 ± 12.97 ^aA^
	26	885	BS	136.277 ± 6.73 ^aABC^	19.566 ± 0.97 ^aA^	155.843 ± 7.48 ^abAB^
			P	100.744 ± 10.31 ^bC^	17.858 ± 2.71 ^aA^	118.602 ± 12.84 ^bB^
			IT	140.466 ± 3.99 ^aABC^	19.874 ± 2.81 ^aA^	160.340 ± 4.7 ^aAB^

Carbon stock in slash pine shoots and roots in plantations of different ages (Site A = 14-year-old; Site B = 24-year-old; Site C = 26-year-old). Year I: from winter 2009 to winter 2010; Year II: from spring 2010 to winter 2011. Treatments: BS = Bark Streak, P = Paste, IT = Intact. Lowercase letters in the column compare treatments within the same site. Uppercase letters in the column compare treatments in the same year within and among sites. Carbon values sharing a letter are not significantly different by the Tukey test (*p* ≤ 0.05).

**Table 5:** Carbon stock in oleoresin biomass harvested from slash pine plantations.

	Site	Site Tree Density (Trees Per ha)	Chemically Induced Slash Pine Oleoresin (MgC.ha^−1^)	Nonchemically Induced Slash Pine Oleoresin (MgC.ha^−1^)
Year I	A	900	3.362 ± 0.16 ^a^	1.660 ± 0.06 ^a^
	B	600	2.095 ± 0.09 ^b^	0.905 ± 0.04 ^b^
	C	900	3.464 ± 0.22 ^a^	1.636 ± 0.08 ^a^
Year II	A	885	2.820 ± 0.13 ^a^	1.827 ± 0.09 ^a^
	B	585	1.757 ± 0.10 ^b^	0.731 ± 0.04 ^c^
	C	885	1.974 ± 0.17 ^b^	1.428 ± 0.08 ^b^

## Data Availability

Not applicable.

## Supplementary Materials

The following supporting information can be downloaded at: https://www.mdpi.com/article/10.3390/biology12020324/s1, Table S1: Physicochemical analysis of samples collected at different soil depths in three slash pine plantations; Figure S1. Height (a) total dry biomass (shoot plus root fractions) (b) and dry biomass separated by treatments (c) of slash pine trees growing at three different plantations. Site A: 14-, Site B: 24-, Site C: 26-year-old slash pine stands (age at the installation of the experiments). Pine trees were felled in 2010 and 2011 (Years I and II, respectively). Lowercase letters compare tree height (a) and weight (c) in different treatments within sites and crop years. In b, lowercase letters compare total dry weight among sites and crop years. Bars sharing a letter are not significantly different by Tukey test (*p* ≤ 0.05). Bars not showing letters indicate no statistical differences among treatments within the same site; Figure S2. Biomass partitioning of slash pine trees growing at three different age plantations. Site A: 14-year-old, site B: 24-year-old, site C: 26-year-old. The percentage was calculated based on the biomass weight (kg) of 45 trees per assessed pine stand (the panel on the tree trunk is not related to the treatment but is merely illustrative; Figure S3. Diameter at breast height (DBH) of slash pine trees submitted to three different treatments. DBHi: initial DBH; DBHf: final DBH (measured at the time when trees were felled). a. Year I (2009–2010); b. Year II (2009–2011). Site A: 14-, Site B: 24-, Site C: 26-year-old slash pine stands (age at the installation of the experiments). * Indicates the significant statistical difference between DBHi and DBHf in the same tree and treatment by one-tailed paired t-test (*p* ≤ 0.05) or Wilcoxon, as appropriate; Figure S4. Lignin content in wood tissues from plants growing at 14-year-old (Site A) and 24-year-old (Site B) slash pine plantations; Figure S5. Total carbon stock average per pine plantation. Site A: 14-, Site B: 24-, Site C: 26-year-old slash pine stands (age at the installation of the experiments). Pine trees were felled in 2010 and 2011 (Years I and II, respectively). Lowercase letters compare tree carbon contents in different sites and years. Bars sharing a letter are not significantly different by Tukey test (*p* ≤ 0.05).
