# Supplementary material for: Carbon Sequestration in Resin-Tapped Slash Pine (Pinus elliottii Engelm.) Subtropical Plantations
Source: Biology (Basel). 2023 Feb 16;12(2):324. doi: 10.3390/biology12020324 (PMC9953639; doi:10.3390/biology12020324)
Supplement: Supplementary file 1 [file biology-12-00324-s001.zip › biology-2120878-supplementary.pdf]

SUPPLEMENTARY MATERIAL

Table S1. Physicochemical analysis of samples collected at different soil depths in three slash pine plantations

Depth →		20 cm			30 cm			60 cm			90 cm		
Site →		A	B	C	A	B	C	A	B	C	A	B	C
**Clay**		7.30±2.31 ^{A}	6.00±0.00 ^{A}	5.00±0.00 ^{A}	10.0±0.00 ^{A}	6.0±0.00 ^{B}	5.00±0.00 ^{C}	10.0±0.00 ^{A}	6.00±0.00 ^{B}	5.00±0.00 ^{C}	17.66±1.15 ^{A}	6.00±0.00 ^{B}	5.00±0.00 ^{C}
**pH**		4.30±0.10 ^{C}	4.90±0.00 ^{B}	5.26±0.06 ^{A}	4.36±0.12 ^{C}	4.86±0.06 ^{B}	5.26±0.06 ^{A}	4.56±0.06 ^{B}	4.80±0.00 ^{A}	5.50±0.00 ^{A}	4.80±0.00 ^{B}	4.80±0.00 ^{B}	5.83±0.06 ^{A}
**O.M.**		2.70±0.26 ^{A}	0.53±0.20 ^{C}	2.06±0.21 ^{B}	1.83±0.12 ^{A}	0.36±0.15 ^{B}	2.13±0.15 ^{A}	1.26±0.50 ^{B}	0.13±0.06 ^{C}	2.30±0.36 ^{A}	0.56±0.06 ^{B*}	0.23±0.06 ^{C*}	2.33±0.23 ^{A*}
**Al**	$cmol \cdot dm^{-3}$	3.30±0.35 ^{A}	0.70±0.10 ^{A}	0.20±0.00 ^{A}	3.00±0.61 ^{A}	0.40±0.00 ^{B}	0.16±0.06 ^{C}	1.56±0.32 ^{A}	0.46±0.06 ^{A}	0.00±0.00 ^{B}	1.10±0.10 ^{A}	0.53±0.06 ^{B}	0.00±0.00 ^{C}
**S**	$mg \cdot dm^{-3}$	12.0±0.00 ^{A}	4.80±0.10 ^{B}	2.06±0.15 ^{C}	11.0±0.00 ^{A}	9.40±1.39 ^{A}	1.63±0.25 ^{B}	8.90±0.35 ^{B}	17.66±1.15 ^{A}	1.93±0.06 ^{C}	27.66±7.64 ^{A}	9.16±0.76 ^{B}	9.43±0.21 ^{B}
**Zn**	$mg \cdot dm^{-3}$	0.27±0.06 ^{B}	0.20±0.00 ^{B}	0.50±0.10 ^{A}	0.13±0.06 ^{B}	0.20±0.00 ^{B}	0.50±0.10 ^{A}	0.10±0.00 ^{C}	0.13±0.06 ^{AB}	0.33±0.06 ^{A}	0.10±0.00 ^{A}	0.13±0.06 ^{AB}	0.36±0.12 ^{A}
**Cu**	$mg \cdot dm^{-3}$	0.10±0.00 ^{C}	0.20±0.00 ^{B}	0.33±0.06 ^{A}	0.10±0.00 ^{B}	0.20±0.00 ^{AB}	0.33±0.06 ^{A}	0.10±0.00 ^{A}	0.20±0.00 ^{A}	0.33±0.06 ^{A}	0.26±0.06 ^{B}	0.20±0.00 ^{B}	0.50±0.10 ^{A}
**B**	$mg \cdot dm^{-3}$	0.63±0.06 ^{A}	0.26±0.10 ^{B}	0.20±0.00 ^{B}	0.50±0.00 ^{A}	0.33±0.06 ^{AB}	0.23±0.06 ^{B}	0.20±0.00 ^{B}	0.43±0.06 ^{A}	0.23±0.06 ^{AB}	0.26±0.06 ^{B}	0.53±0.06 ^{AB}	0.20±0.00 ^{C}
**Mn**	$mg \cdot dm^{-3}$	1.00±0.00 ^{A}	1.66±1.20 ^{A}	4.00±1.73 ^{A}	1.00±0.00 ^{B}	1.00±0.00 ^{B}	3.33±0.58 ^{A}	1.00±0.00 ^{A}	1.33±0.58 ^{A}	2.00±0.00 ^{A}	1.00±0.00 ^{A}	1.00±0.00 ^{A}	1.66±0.58 ^{A}
**Fe**	$mg \cdot dm^{-3}$	2.30±0.79 ^{A}	0.30±0.00 ^{B}	0.23±0.06 ^{B}	2.16±0.64 ^{A}	0.20±0.00 ^{B}	0.20±0.00 ^{B}	1.06±0.15 ^{A}	0.16±0.06 ^{B}	0.20±0.10 ^{B}	0.56±0.12 ^{A}	0.13±0.06 ^{B}	0.20±0.00 ^{B}
**Al+H**	$cmol \cdot dm^{-3}$	17.0±4.10 ^{A}	2.13±0.10 ^{B}	1.33±0.12 ^{B}	24.73±9.50 ^{A}	1.80±0.17 ^{B}	1.33±0.12 ^{B}	9.76±1.10 ^{A}	1.80±0.17 ^{B}	1.23±0.15 ^{B}	7.86±1.76 ^{A}	2.16±0.57 ^{B}	1.90±0.17 ^{B}
**CEC**	$cmol \cdot dm^{-3}$	17.5±4.19 ^{A}	2.40±0.20 ^{B}	2.43±0.06 ^{B}	25.26±9.60 ^{A}	2.06±0.12 ^{B}	2.46±0.06 ^{B}	10.9±1.15 ^{A}	2.10±0.10 ^{B}	2.26±0.35 ^{B}	10.6±2.36 ^{A}	2.40±0.53 ^{B}	2.73±0.12 ^{B}
**Al**	% SAT of CEC	86.2±0.40 ^{A}	70.6±8.60 ^{B}	15.16±0.72 ^{C}	86.23±2.10 ^{A}	61.60±1.78 ^{B}	12.53±3.93 ^{C}	57.8±7.36 ^{A}	63.53±7.84 ^{A}	0.00±0.00 ^{B}	67.4±3.30 ^{A}	29.46±6.36 ^{B}	0.00±0.00 ^{C}
**Ca/Mg**		1.30±0.29 ^{A}	1.33±0.60 ^{A}	1.33±0.42 ^{A}	1.50±0.50 ^{A}	1.00±0.00 ^{A}	1.20±0.00 ^{A}	0.70±0.00 ^{A}	1.33±0.58 ^{A}	1.26±0.23 ^{A}	0.6±0.35 ^{A}	1.00±0.00 ^{A}	1.70±0.00 ^{A}
**Ca/K**	RATIO	4.46±2.20 ^{B}	2.40±0.00 ^{B}	12.33±1.53 ^{A}	3.36±1.56 ^{B}	2.26±0.81 ^{B}	12.66±0.58 ^{A}	9.43±5.36 ^{A}	4.36±2.32 ^{A}	15.33±6.43 ^{A}	10.33±2.89 ^{A}	1.90±0.44 ^{B}	11.0±0.00 ^{A}
**Mg/K**		3.30±1.21 ^{B}	2.00±0.70 ^{B}	9.66±1.53 ^{A}	2.20±0.36 ^{B}	2.26±0.81 ^{B}	10.66±0.58 ^{A}	13.66±7.51 ^{A}	3.13±0.47 ^{A}	11.66±3.21 ^{A}	21.33±12.42 ^{A}	1.90±0.44 ^{B}	6.66±0.58 ^{AB}

Site **A**: 14-, Site **B**: 24-, Site **C**: 26-year-old plantations. O.M.: organic matter. Al: aluminum; S: sulfur; Zn: zinc; Cu: copper; B: boron; Mn: manganese; Fe: iron; H: hydrogen; Ca: calcium; Mg: magnesium; CEC: cation exchange capacity. Clay content was determined by densitometry; pH was determined in water (1:1); P and K were determined by Mehlich method I.; O.M. was determined by moist digestion; exchangeable Ca, Mg, Al, and Mn were extracted with KCl 1.0 $mol \cdot L^{-1}$; S-SO_4 was extracted with $CaHPO_4$ 500 $mg \cdot L^{-1}$ of P; Zn and Cu were extracted with HCl 0.1 L^{-1}; B was extracted with hot water. Cm = centimole. Each mean was calculated with ten combined samples from the same soil layer. Values sharing a letter (within the same depth) are not significantly different by Tukey test ($p \leq 0.05$).

a

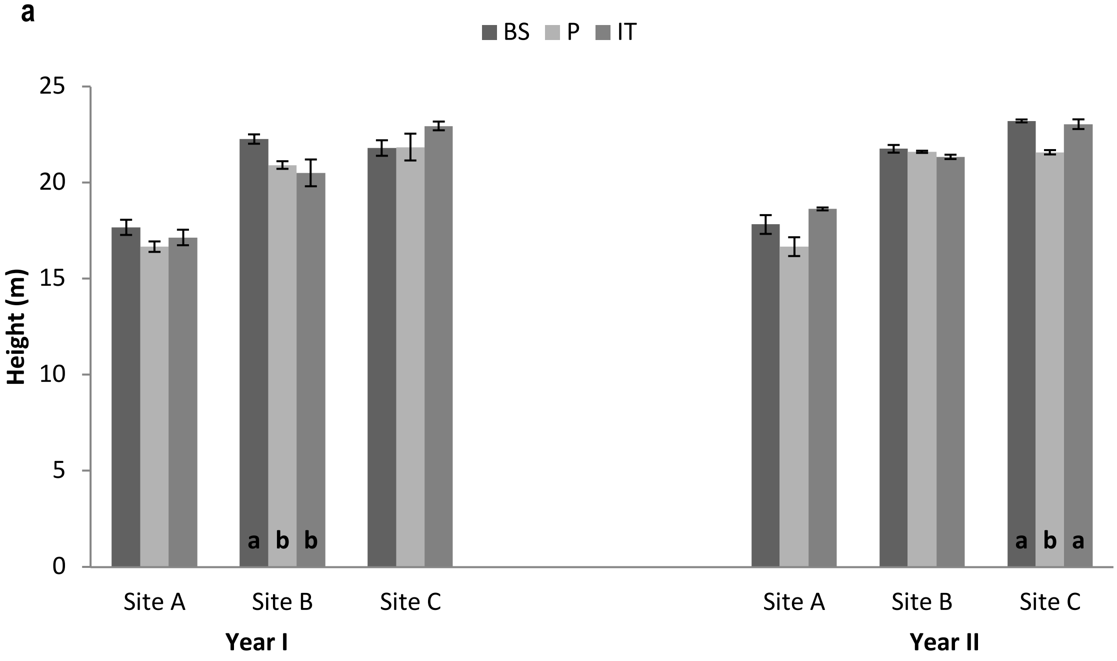

BS P IT
25
20
15
10
5
0
Height (m)
a b b
a b a
Site A
Site B
Site C
Year I
Site A
Site B
Site C
Year II

b

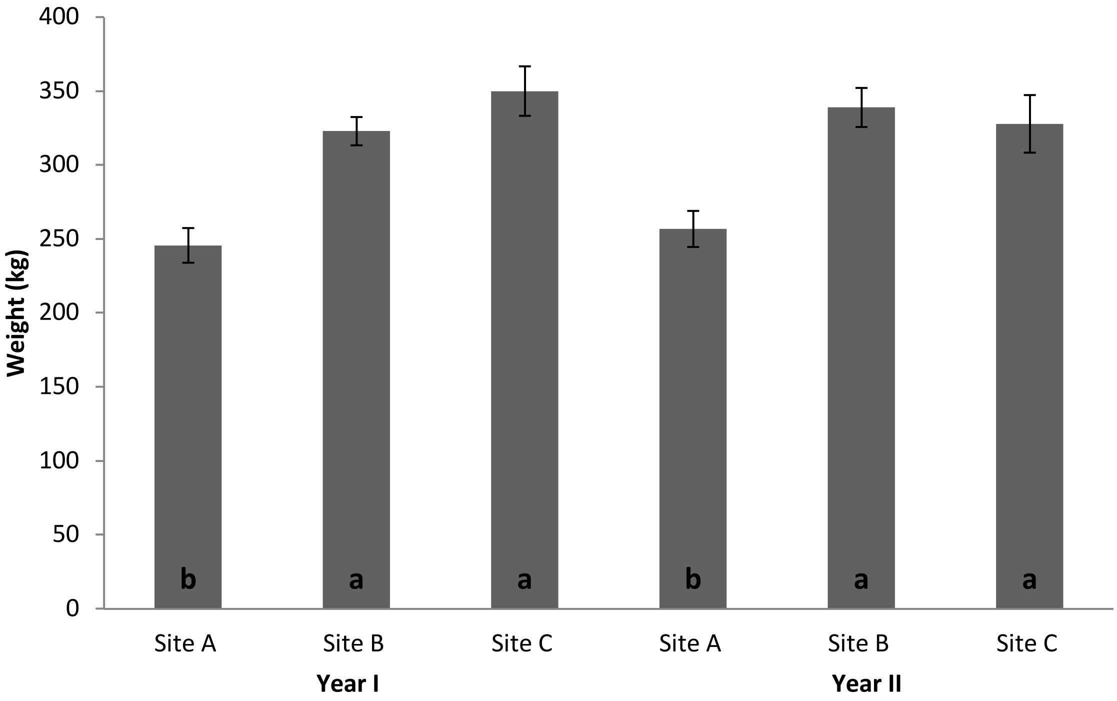

400
350
300
250
200
150
100
50
0
Weight (kg)
b a a b a a
Site A
Site B
Site C
Site A
Site B
Site C
Year I
Year II

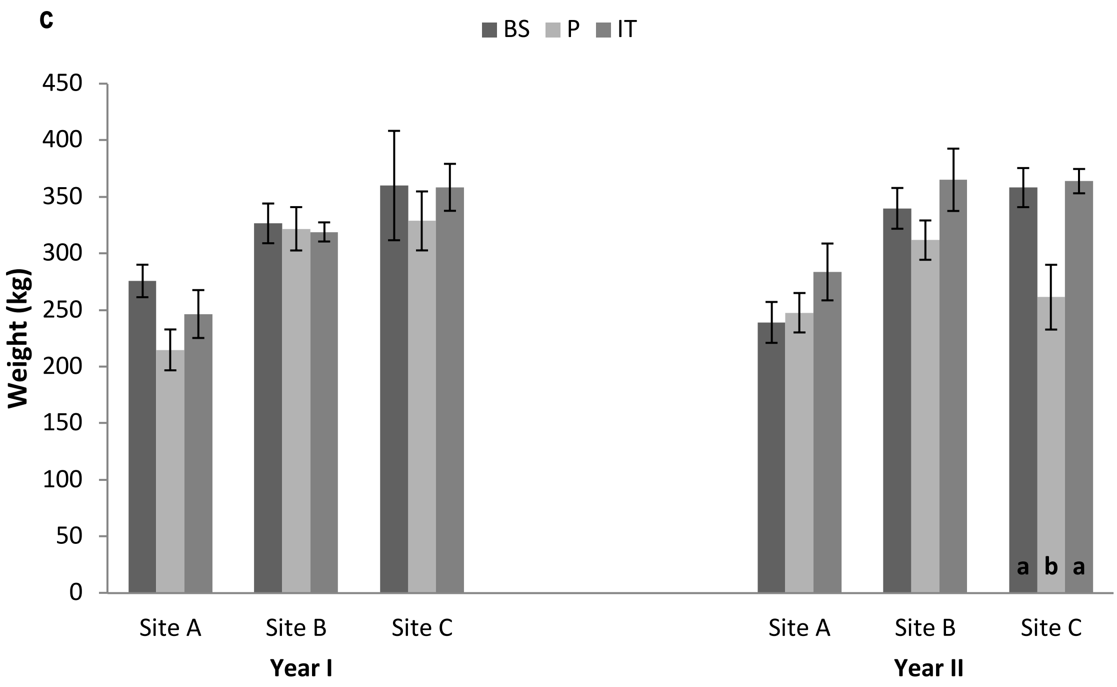

Figure S1. Height (**a**), total dry biomass (shoot plus root fractions) (**b)** and dry biomass separated by treatments **(c)** of slash pine trees growing at three different plantations. Site **A**: 14-, Site **B**: 24-, Site **C**: 26-year-old slash pine stands (age at the installation of the experiments). Pine trees were felled in 2010 and 2011 (Years I and II, respectively). Lowercase letters compare tree height (**a**) and weight (**c**) in different treatments within sites and crop year. In **b**, lowercase letters compare total dry weight among sites and crop years. Bars sharing a letter are not significantly different by Tukey test ($p \leq 0.05$). Bars not showing letters indicate no statistical differences among treatments within the same site.

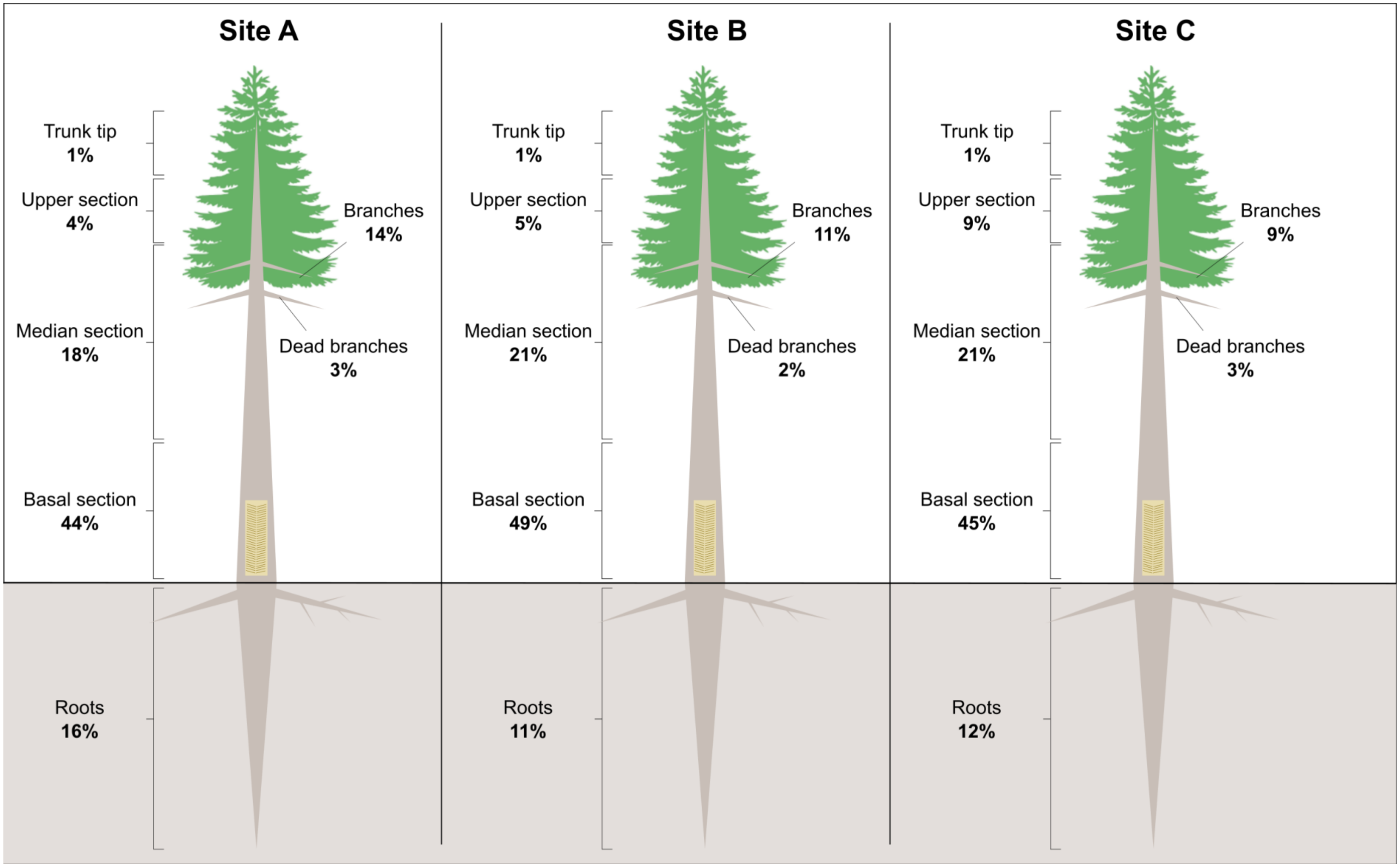

Figure S2. Biomass partitioning of slash pine trees growing at three different age plantations. Site **A**: 14-year-old, site **B**: 24-year-old, site **C**: 26-year-old. The percentage was calculated based on biomass weight (kg) of 45 trees per assessed pine stand (the panel on the tree trunk is not related to the treatment but merely illustrative).

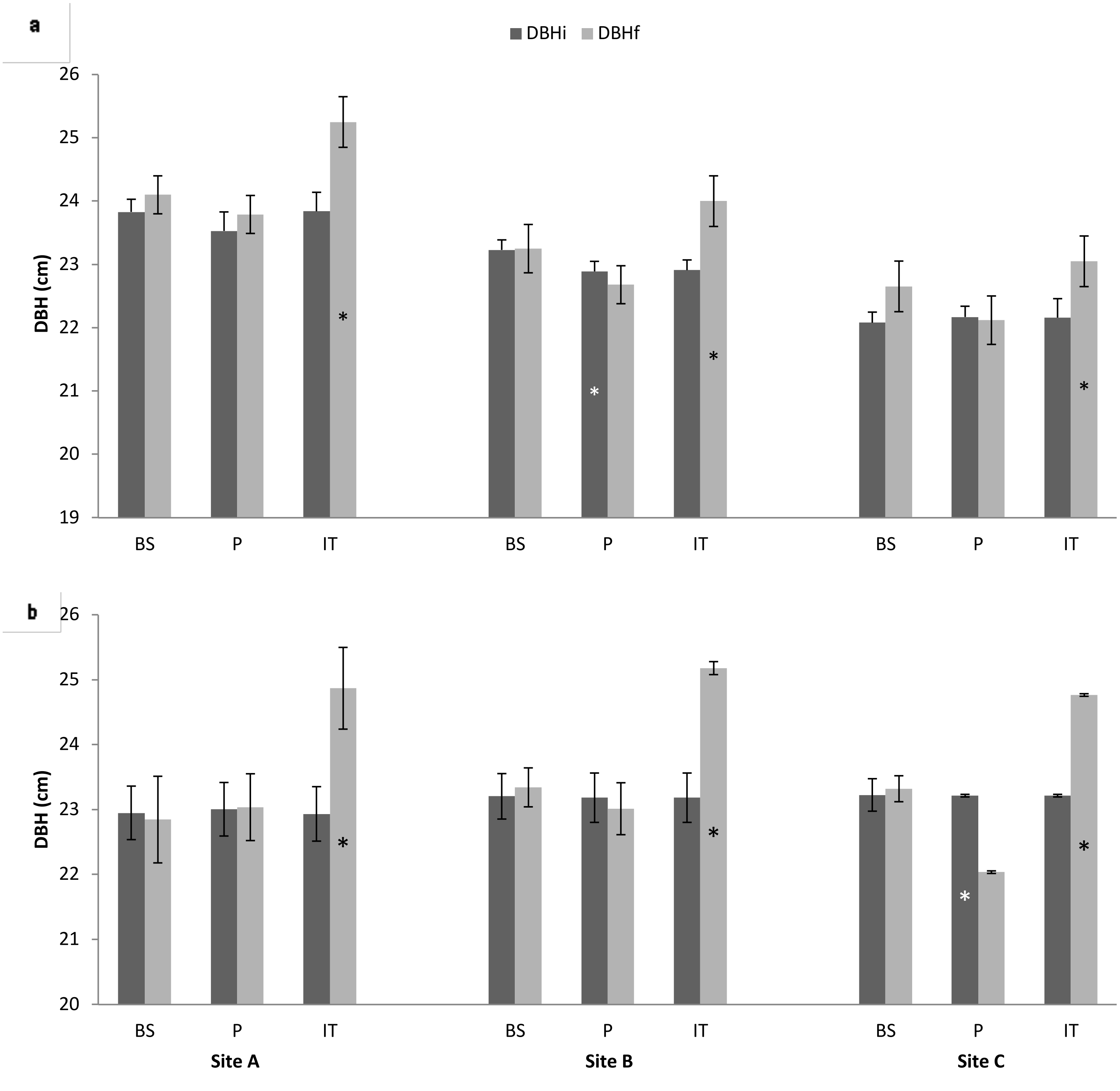

Figure S3. Diameter at breast height (**DBH**) of slash pine trees submitted to three different treatments. **DBHi**: initial DBH; **DBHf**: final DBH (measured at the time when trees were felled). **a.** Year I (2009-2010); **b.** Year II (2009-2011). Site A: 14-, Site B: 24-, Site C: 26-year-old slash pine stands (age at the installation of the experiments). * Indicates significant statistical difference between DBHi and DBHf in the same tree and treatment by one-tailed paired t-test ($p \leq 0.05$) or Wilcoxon, as appropriate.

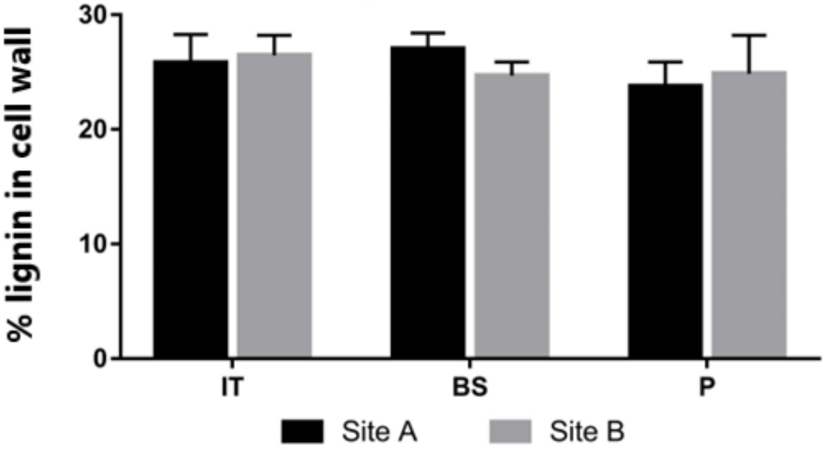

Figure S4. Lignin content in wood tissues from plants growing at 14-year-old (Site A) and 24-year-old (Site B) slash pine plantations.

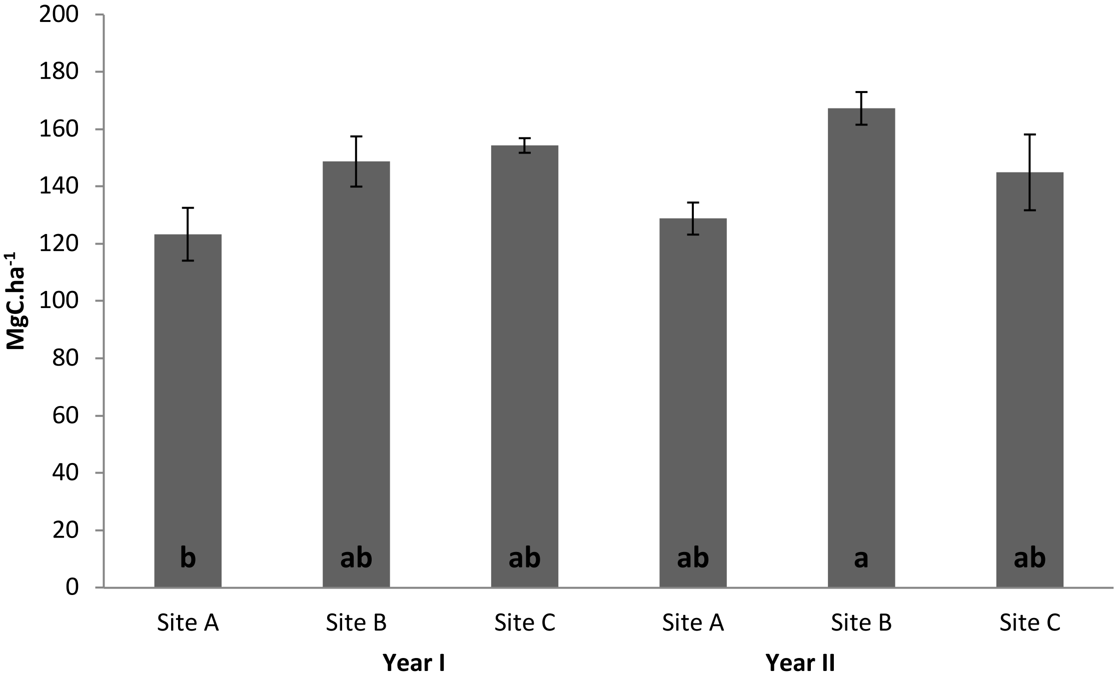

Figure S5. Total carbon stock average per pine plantation. Site **A**: 14-, Site **B**: 24-, Site **C**: 26-year-old slash pine stands (age at the installation of the experiments). Pine trees were felled in 2010 and 2011 (Years I and II, respectively). Lowercase letters compare tree carbon contents in different sites and years. Bars sharing a letter are not significantly different by Tukey test ($p \leq 0.05$).
